# Overexpression of *Solanum habrochaites* microRNA319d (sha-miR319d) confers chilling and heat stress tolerance in tomato (*S. lycopersicum*)

**DOI:** 10.1186/s12870-019-1823-x

**Published:** 2019-05-23

**Authors:** Xiaopu Shi, Fangling Jiang, Junqin Wen, Zhen Wu

**Affiliations:** College of Horticulture, Nanjing Agricultural University, Key Laboratory of Horticultural Plant Biology and Germplasm Innovation in East China, Ministry of Agriculture, Nanjing, 210095 People’s Republic of China

**Keywords:** miR319, *Solanum habrochaites*, Tomato, Chilling stress, Heat stress

## Abstract

**Background:**

MicroRNA319 (miR319) acts as an essential regulator of gene expression during plant development and under stress conditions. Although the role of miR319a in regulating leaf development has been well studied in tomato (*Solanum lycopersicum*), the function of the recently discovered wild tomato *Solanum habrochaites* miRNA319d (sha-miR319d) remains poorly understood. In this study, we overexpressed sha-miR319d in cultivated tomato ‘Micro-Tom’ to further investigate its role in tomato temperature stress responses.

**Results:**

Under chilling or heat stress, sha-miR319d-overexpressing plants showed enhanced stress tolerance, including lower relative electrolyte leakage (REL), malondialdehyde (MDA) concentration, O_2_^−^ generation and H_2_O_2_ concentration and higher chlorophyll contents and Fv/Fm values than wild-type (WT) plants. Overexpression of sha-miR319d enhanced the activities of superoxide dismutase (SOD) and catalase (CAT), with possible correlation with elevated expression levels of the genes *FeSOD, CuZnSOD* and *CAT*. Moreover, different expression levels of key genes involved in chilling (*MYB83* and *CBF1*), heat (*HsfA1a*, *HsfA1b* and *Hsp90*), and reactive oxygen species (ROS) (*ZAT12* and *ZAT10*) signaling in transgenic plants and WT were determined, suggesting a role for sha-miR319d in regulating tomato temperature stress via chilling, heat and ROS signaling. Silencing *GAMYB-like1* increased tomato chilling tolerance as well as the expression levels of *CBF1*, *CuZnSOD*, *CAT*, *APX1*, *APX2*, *ZAT12* and *ZAT10*. Additionally, overexpression of sha-miR319d in tomato caused plant leaf crinkling and reduced height.

**Conclusions:**

Overexpression of sha-miR319d confers chilling and heat stress tolerance in tomato. Sha-miR319d regulates tomato chilling tolerance, possibly by inhibiting expression of *GAMYB-like1* and further alters chilling, heat and ROS signal transduction. Our research provides insight for further study of the role of sha-miR319d in tomato growth and stress regulation and lays a foundation for the genetic improvement of tomato.

**Electronic supplementary material:**

The online version of this article (10.1186/s12870-019-1823-x) contains supplementary material, which is available to authorized users.

## Background

In plants, microRNAs (miRNAs), single-stranded non-coding RNA molecules with a length of 19–24 nucleotides, serve as gene expression regulators of target messenger RNAs (target mRNAs) at the posttranscriptional level [1, 2]. Increasing evidence suggests that miRNAs play a vital role in plant developmental growth as well as in the adaptation to various stress conditions [3–8].

The miR319 families comprises one of the most ancient and conserved plant miRNA families, and an increasing number of studies have shown that miR319 is involved in the regulation of plant tolerance to stress. In *Arabidopsis*, expression of miR319 is induced by cold stress but is reduced by drought and saline stress [9]. Moreover, high expression levels of OsamiR319 in transgenic rice (*Oryza sativa*) and creeping bentgrass (*Agrostis stolonifera*) increased plant tolerance to salinity and drought stress [10, 11], and OsamiR319-overexpressing lines showed enhanced chill tolerance [7, 12]. The functions of miR319 in plant growth have been well studied. For instance, *jaw-D* and *cin* mutants, in which the loss-of-function of miR319-regulated TEOSINTE BRANCHED/CYCLOIDEA/PROLIFERATING CELL FACTORS (TCPs) occurs, have abnormal, crinkled leaves [5, 13]. In addition, it has been suggested that the enhanced stress tolerance of miR319-overexpressing lines may be explained, at least in part, by morphological changes in leaves [12].

Chilling and heat stress have negative effects on plant growth and reduce crop production. To cope with these extreme temperatures, plants have their own precise regulating mechanism. The irreplaceable transcription factors involved in the chilling signaling pathway, i.e., C-repeat binding factors (CBFs), which are also known as dehydration-responsive element-binding protein 1 s (DREB1s), have been relatively well studied in model plants [14]. An elevated *CBF* expression level is often correlated with higher chilling tolerance [14–16], and the RNAi lines of *CBF1*, *CBF3*, and *cbf123* triple mutants are highly sensitive to chilling stress [17–19]. Wang et al. [7] reported that overexpression of OsamiR319b led to higher expression levels of *DREB1,* which suggested that OsamiR319b may play a role in chilling signal transduction.

With respect to heat stress, the ‘master regulators’ heat shock transcription factors (Hsfs) and heat shock protein (Hsp) are regarded as important components of the heat stress response [20–22]. Under heat stress, plant Hsf and Hsp respond to stress by changing their levels of transcription or protein stability [20, 22, 23]. In addition, in the process of miRNA biosynthesis, changes in the AGO1 conformation, the removal of passenger strands in the miRNA/miRNA* double chain complex, and the formation of the RISC silencing complex all require the participation of Hsp90 dimers [24]. These results suggest a potential role for Hsp and Hsf in heat stress tolerance and miRNA biosynthesis. Several studies showed that miRNAs can mediate the heat stress response by regulating the activity of Hsfs [25–27]. While the heat-induced expression of miR319 has been reported in several species [28, 29], the involvement of miR319 in heat stress regulation is still unknown.

Reactive oxygen species (ROS), which are regarded as stress signaling molecules [22], can be produced as a result of both chilling and heat stress [30, 31]. Chilling stress-response genes also participate in the oxidative stress response [32]. ROS levels in plants also affect expression of *Hsf*s and *Hsps*, which respond to heat stress [33–35]. The ROS signaling transcription factors ZAT12 and ZAT10 also participate in temperature stress regulation [14, 22]. All of these findings suggest a combination of temperature signals and ROS signal transduction. However, it is not clear whether the role of tomato miR319 in temperature stress also occurs via the ROS pathway.

Tomato (*Solanum lycopersicum*) is an important vegetable and is widely grown worldwide. The wild tomato genotype, *S. habrochaites* ‘LA1777’, has a higher chill-tolerant ability than cultivated tomato [36–38]. Our previous study showed that sha-miR319d, a recently discovered member of miR319 from *S. habrochaites*, was strongly induced by both low and high temperatures [29, 39]. The up-regulated expression of miR319d in the temperature stress response is present in various tomato genotypes (unpublished observations). These results suggest a potential role for sha-miR319d in tomato temperature tolerance regulation. However, how sha-miR319d functions in the ability of tomato to resist chilling and heat stress remains unknown.

MiRNAs participate in biological processes by regulating expression of target genes. Several target genes of miR319 have been identified by degradome sequencing, bioinformatics prediction and 5’RACE validation [29, 39, 40]. *GAMYB-like1* is one of the target genes of miR319 in tomato identified by the above methods [29, 40]. Furthermore, *GAMYB-like1* was identified as the target gene of stu-miR319b in potato, and stu-miR319b and sha-miR319d had identical sequences [41]. In addition, our previous experimental results showed that the expression level of *GAMYB-like1* was decreased in *S. habrochaites* ‘LA1777’ under chilling stress, corresponding to an increase in sha-miR319d expression. However, it remains unknown whether sha-miR319d functions in the regulation of *GAMYB-like1* to resist temperature stress.

Here, we overexpressed sha-miR319d in the cultivated tomato ‘Micro-Tom’ and further investigated its effects on the physiological and molecular changes of tomato under temperature stress with the aim of revealing the regulatory mechanism of sha-miR319d under temperature stress from the perspectives of temperature and the ROS signaling pathway. Silencing of the *GAMYB-like1* gene by virus-induced gene silencing (VIGS) has been performed to uncover the effects of *GAMYB-like1*-silencing on tomato under temperature stress conditions and to further explore the regulatory mechanism of sha-miR319d from the point of the target gene. Our results reveal a positive role for sha-miR319d in chilling and heat stress tolerance regulation, with miR319 family members being responsible for the regulation of plant responses to temperature stress. These findings provide a foundation for utilizing sha-miR319d in future genetic improvement of tomato.

## Results

### Sequence analysis of sha-miR319d

The sequence of sha-miR319d is consistent with that of potato (*S. tuberosum*) stu-miR319b (Additional file 1: Figure S1A). In our previous study, the precursor of sha-miR319d (sha-MIR319d) was cloned (GenBank accession number MH230181). Using the sequence of sha-MIR319d, we searched its homologs using miRBase 22.0 [42]. Phylogenetic analysis was performed to reveal the genetic homology between sha-MIR319d and other species’ miR319 precursors (Additional file 1: Figure S1B). Potato (*S. tuberosum*) stu-MIR319b showed the highest homology with sha-MIR319d (Additional file 1: Figure S1B).

### Prediction and expression analysis of sha-miR319d target genes

Using the psRNA Target tool [43], we obtained several potential targets of sha-miR319d, including *TCP1*, *TCP2*, *TCP3*, *TCP10*, *TCP24*, *TCP29*, *GAMYB-like1*, protein of unknown function DUF761 coding gene (*DUF761*, *Solyc02g079010.2*), Kelch domain-containing protein 3 coding gene (*Kelch-type3*, *Solyc11g 007960.1*) and pentatricopeptide repeat-containing protein coding gene (*PPR-containing*, *Solyc08g 069010.2*). The complementary area between targets and sha-miR319d is shown in Additional file 2: Figure S2A. In addition, the expression levels of putative target genes were markedly suppressed in sha-miR319d-overexpressing plants (Additional file 2: Figure S2B).

### Overexpression of sha-miR319d affects leaf morphogenesis and plant height

**Fig. 1:**
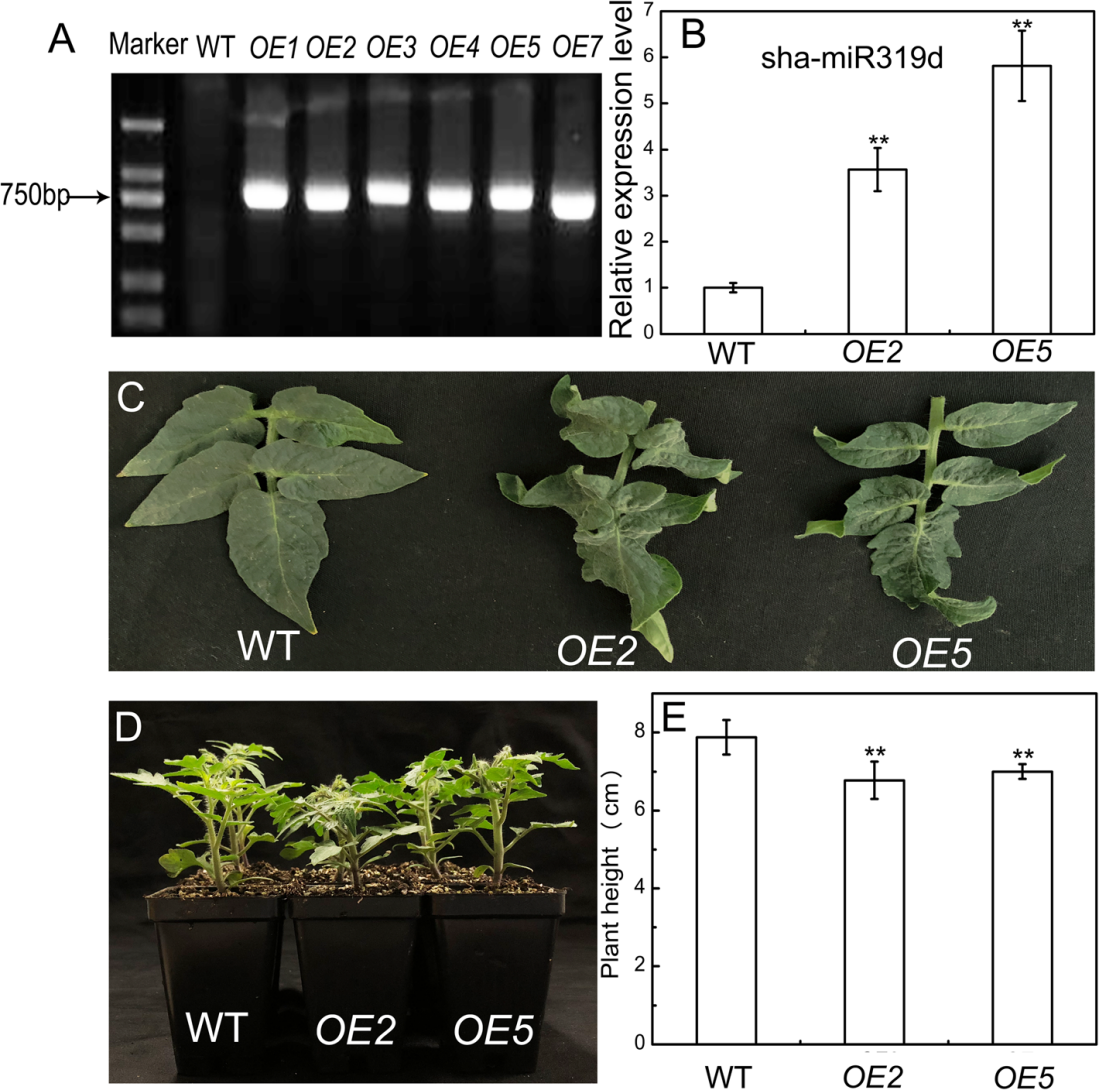
Identification of sha-miR319d overexpression transgenic plants. **a** Genomic PCR analysis of sha-MIR319d in WT and transgenic plants. **b** Expression levels of sha-miR319d in WT and T2 generations of transgenic plants. The reference gene for sha-miR319d was *U6snRNA*. Each value was the mean of three biological repeats ± the standard deviation (SD). **c** Leaf morphogenesis of WT and transgenic lines (T1 generations). **d, e** The plant height of 30-day-old WT and transgenic lines (T2 generations). Asterisks indicate significant differences between WT and transgenic plants. *, *P* < 0.05; **, *P* < 0.01, Student’s t test

To investigate the biological function of sha-miR319d, we overexpressed sha-miR319d and transformed it into tomato ‘Micro-Tom’. Genomic PCR was used to identify the positive transformants using CaMV35S forward plus gene-specific reverse primer pairs. In total, 14 individual transgenic lines (T0 generation) were obtained, and 12 of them were positive transformants. Several identification results are shown in Fig. 1a. Obvious bands were detected in transgenic lines (*OE1, OE2, OE3, OE4, OE5,* and *OE7*), while no bands were detected in WT. Considering the amount of seeds of transgenic lines, two independent lines, *OE2* and *OE5,* were selected for subsequent experiments. The expression level of sha-miR319d in T1 transgenic generations *OE2* and *OE5* is shown in Additional file 3 Figure S3A. The T2 transgenic generations of *OE2* and *OE5* still showed higher expression levels than did WT plants (Fig. 1b).

**Fig. 2:**
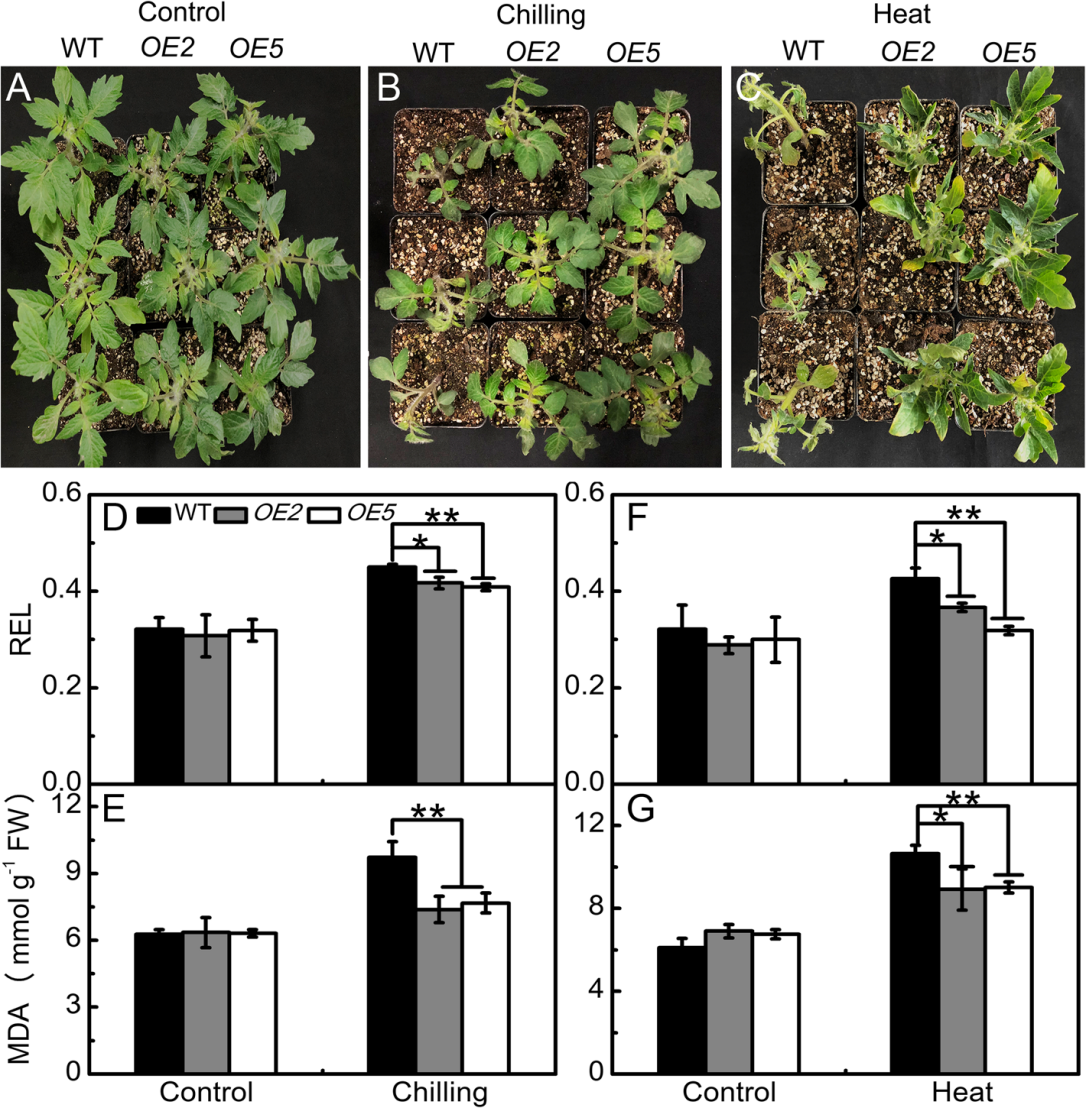
The effect of overexpression of sha-miR319d on plant growth under control and temperature-stress conditions. **a** Phenotypes of WT and transgenic lines (T2 generations) under control (25 °C) conditions. **b** Phenotypes of WT and transgenic lines (T2 generations) under chilling (4 °C) conditions for 5 d. **c** Phenotypes of WT and transgenic lines (T2 generations) under heat (40 °C) conditions for 5 d. **d, e** Effects of chilling stress on REL (D) and the MDA content (**f**) in WT and transgenic lines (T2 generations). **f, g** Effects of heat stress on REL (F) and MDA (**g**). The data in (**d**-**g**) are the means of three biological repeats ± the standard deviation (SD). Asterisks indicate significant differences between transgenic lines and WT. *, *P* < 0.05; **, P < 0.01, Student’s t test

Under normal conditions, sha-miR319d overexpression lines had curly leaves, and curved morphogenesis was obviously observed in mature plants (approximately 75 days old) (Fig. 2c). Another phenotype of transgenic lines was the lower plant height (Fig. 2d). Approximately 30-day-old transgenic plants *OE2* and *OE5* were 6.77 cm and 7.00 cm tall, respectively (Fig. 2e), which were significantly shorter in comparison with WT (7.87 cm). The curly leaf phenotypes and suppressed plant height were also observed in T1 generations (Additional file 3: Figure S3B). These results suggest the potential role of sha-miR319d in plant growth.

### Overexpression of sha-miR319d increases tomato chilling and heat tolerance

#### Phenotypes of transgenic lines under temperature stress

When 30-day-old transgenic lines and WT seedlings were subjected to chilling stress (4 °C) for 5 d, all plants showed wilting symptoms, whereas the WT leaves showed more severe withering than those of transgenic plants *OE2* and *OE5* (Fig. 2b). Increased heat tolerance of transgenic lines was also observed when 30-day-old seedlings were subjected to heat stress (40 °C). After 40 °C treatment for 5 d, *OE2* and *OE5* showed fewer withering symptoms than WT.

#### Decreased levels of relative electrolyte leakage (REL) and malondialdehyde (MDA) in sha-miR319d-overexpressing lines

There were no differences in the REL and MDA contents between WT and transgenic plants under control conditions (25 °C). However, the RELs of *OE2* and *OE5* were 0.41 and 0.40 lower, respectively, than that (0.45) of WT after chilling treatment for 1 d (Fig. 2d). Accordingly, the amount of MDA in *OE2* and *OE5* increased 1.16- and 1.21-fold under chilling stress compared to normal conditions, whereas a 1.55-fold increase was observed in WT (Fig. 2e).

REL and MDA contents under heat treatment were also measured. As shown in Fig. 2f and g, after heat treatment for 1 d, REL and MDA contents in transgenic lines were less increased than those in WT. The phenotypes of T1 generations under chilling or heat stress are shown in Additional file 2, and transgenic plants showed fewer injuries than WT plants.

#### Overexpression of sha-miR319d increases chlorophyll contents and maximum quantum efficiency of photosystem II (Fv/Fm) under chilling and heat stress

**Fig. 3:**
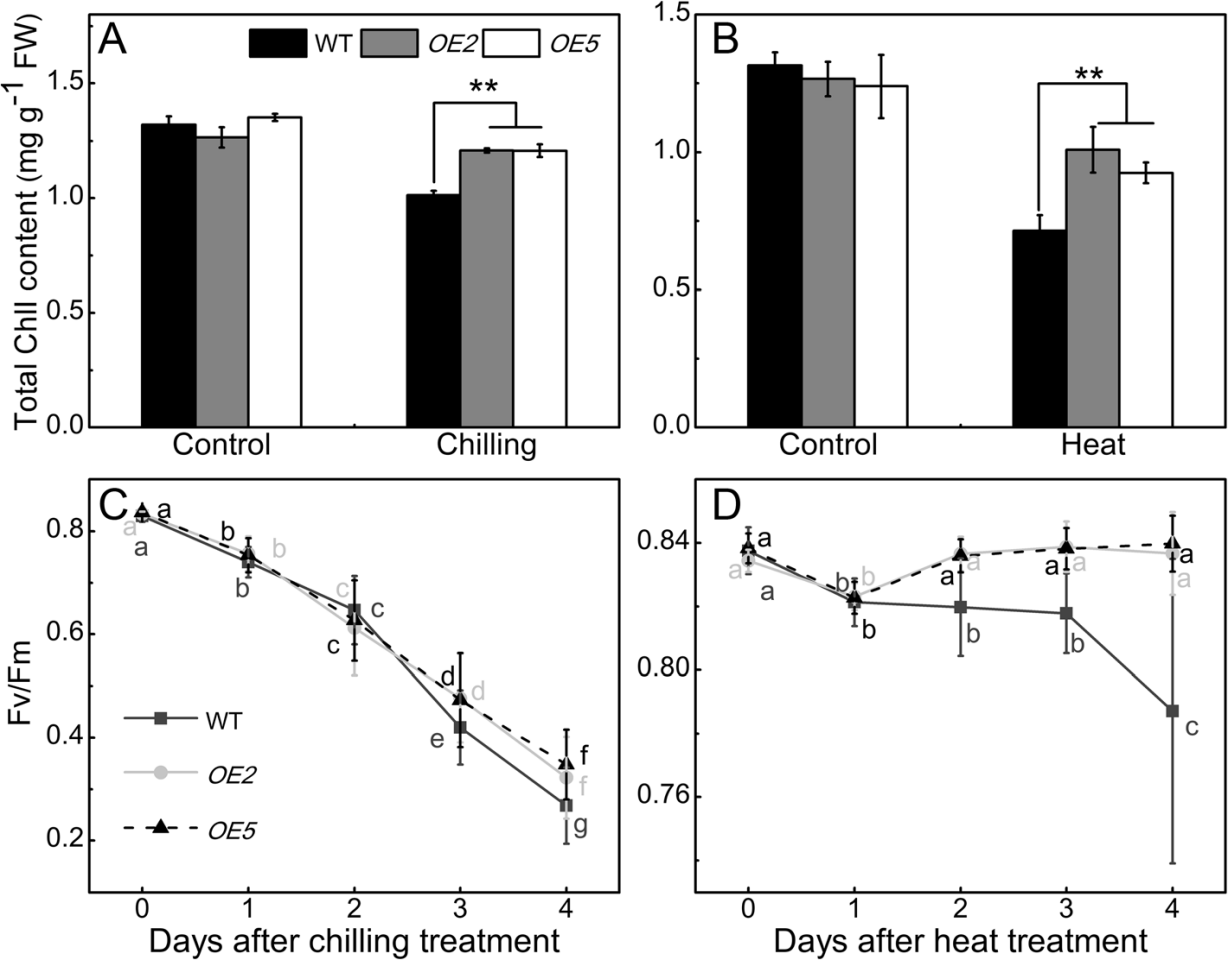
The effect of overexpression of sha-miR319d on the chlorophyll content and Fv/Fm. **a** Chlorophyll content of WT and transgenic plants under control or chilling (4 °C) treatment for 5 d. **b** Chlorophyll content of WT and transgenic plants under control or heat (40 °C) treatment for 5 d. **C** Fv/Fm of WT and transgenic plants under chilling (4 °C) treatment. **d** Fv/Fm of WT and transgenic plants under heat (40 °C) treatment. T2 generations of transgenic plants as well as the WT were used for measurements of Fv/Fm and the chlorophyll content. The data in (**a**) and (**b**) are the mean of three biological repeats ± the standard deviation (SD). Asterisks indicate significant differences between transgenic lines and WT. *, *P* < 0.05; **, *P* < 0.01, Student’s t test. The data in (**c**) and (**d**) are the means ± the standard deviation (SD) obtained from 24 seedlings, and letters above indicate the statistical significance of differences (P < 0.05) analyzed by variance (ANOVA) using SPSS 18.0

There was no obvious difference in the amount of chlorophyll in WT and transgenic plants under control conditions; however, transgenic plants showed higher chlorophyll contents compared with WT after 5 d of chilling or heat treatment (Fig 3a and b). There was no significant difference in Fv/Fm between WT and *OE* lines under the control conditions (0 d of treatment) (Fig. 3c and d). Fv/Fm showed a similar decreasing trend in WT and transgenic plants during chilling treatment and was significantly lower in the WT (0.42, 0.27) than in *OE2* (0.47, 0.32) and *OE5* (0.47, 0.35) after chilling stress (3 d, 4 d, respectively) (Fig. 3c). By 4 d after heat treatment, the Fv/Fm of WT plants was markedly lower than that of *OE* lines (Fig. 3d). These results suggest that sha-miR319d overexpression decreases temperature stress-induced PSII photoinhibition.

#### Overexpression of sha-miR319d reduced O_2_^−^ and H_2_O_2_ accumulation under chilling and heat stress

**Fig. 4:**
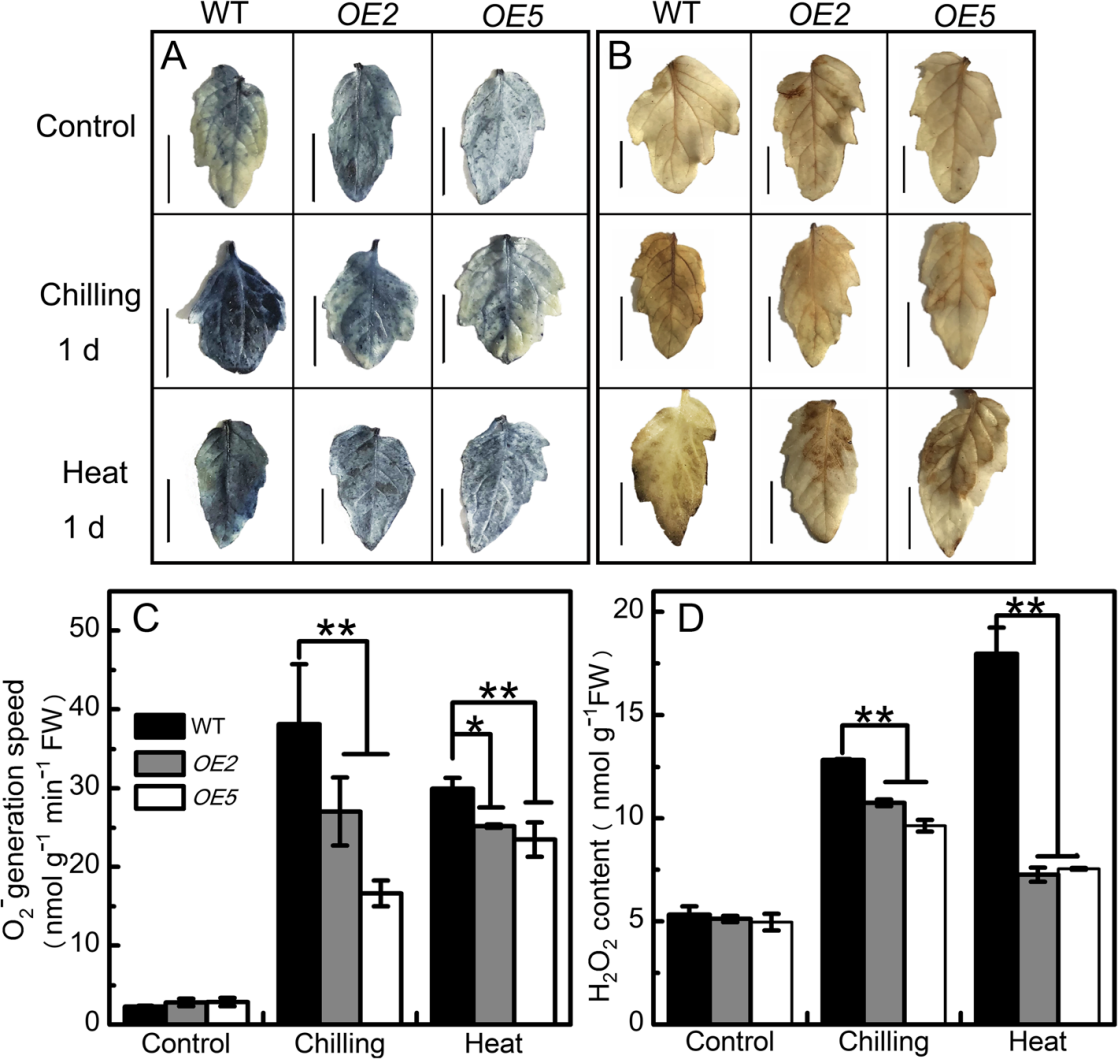
The effect of overexpression of sha-miR319d on ROS accumulation. NBT staining for O_2_^−^ in the leaves of WT and transgenic plants under control conditions and after 1 d of chilling (4 °C) or heat (40 °C) treatment. **b** DAB staining for H_2_O_2_ in the leaves of WT and transgenic plants under control conditions and after 1 d of chilling (4 °C) or heat (40 °C) treatment. **c** Quantitative analysis of O_2_^−^ in the leaves of WT and transgenic plants under control conditions and after 1 d of chilling (4 °C) or heat (40 °C) treatment. **d** Quantitative analysis of H_2_O_2_ in the leaves of WT and transgenic plants under control conditions and after 1 d of chilling (4 °C) or heat (40 °C) treatment. T2 generations of transgenic plants as well as WT were used for O_2_^−^ and H_2_O_2_ histochemical staining and quantitative analysis. Each value is the mean of three biological repeats ± the standard deviation (SD). Asterisks indicate significant differences between transgenic lines and WT. *, *P* < 0.05; **, *P* < 0.01, Student’s t test. The bar in (**a**) and (**b**) indicates 1 cm

The O_2_^−^ and H_2_O_2_ contents were determined using nitro blue tetrazolium (NBT) and 3′, 3′- diaminobenzidine [44] staining, respectively. Under control conditions, a few small blue spots that indicated the polymerization product of O_2_^−^ were distributed on the leaves of both WT and transgenic lines, and no visible difference was observed (Fig. 4a). The light brown polymerization product of H_2_O_2_ appeared only in the veins of leaves of the WT and transgenic plants (Fig. 4b). After chilling or heat stress, staining was stronger in both the WT and transgenic lines compared with the control group. The intensities of blue and brown spots in the WT leaves were stronger than those of transgenic lines (Fig. 4a and b), indicating that ROS accumulation in transgenic lines was lower than that in the WT after 1 d of chilling or heat stress. Similar NBT and DAB staining results were observed in T1 generations after temperature stress (Additional file 4: Figure S4A and B). We also performed quantitative analysis of O_2_^−^ and H_2_O_2_ to verify the histochemical staining (Fig. 4c and d), and similar staining results were observed in T1 generations. These results revealed that sha-miR319d overexpression reduced O_2_^−^ and H_2_O_2_ accumulation.

#### Overexpression of sha-miR319d causes changes in antioxidant enzymes under chilling and heat stress

**Fig. 5:**
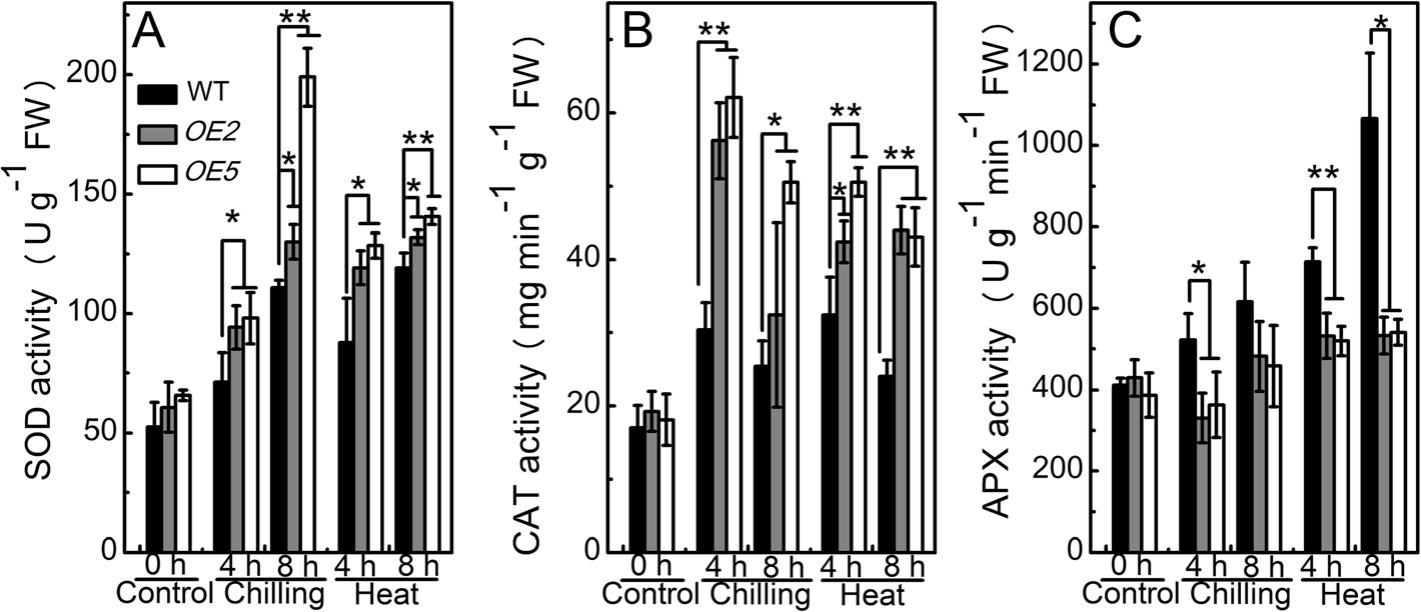
The effect of overexpression of sha-miR319d on the activities of antioxidant enzymes. **a** SOD activities of WT and transgenic plants under control, chilling stress (4 °C) and heat stress (40 °C) conditions. **b** CAT activities of WT and transgenic plants under control, chilling stress (4 °C) and heat stress (40 °C) conditions. **c** APX activities of WT and transgenic plants under control, chilling stress (4 °C) and heat stress (40 °C) conditions. T2 generations of transgenic plants as well as WT were used. Each value is the mean of three biological repeats ± the standard deviation (SD). Asterisks indicate significant differences between transgenic lines and WT. *, *P* < 0.05; **, *P* < 0.01, Student’s t test.

Under normal conditions, the activities of superoxide dismutase (SOD, EC 1.15.1.1) and catalase (CAT, EC 1.11.1.6), and ascorbate peroxidase (APX, EC 1.11.1.11) in WT and transgenic plants were almost at the same level (Fig. 5). The SOD activities in *OE2* and *OE5* were 94.2 and 98.0 U g^− 1^ FW, respectively, whereas that in the WT was 87.8 U g^− 1^ FW after 4 h of chilling treatment (Fig. 5a). After 8 h after chilling treatment, the SOD activities in *OE2* and *OE5* were still markedly higher than those in the WT (Fig. 5a). The CAT activity increased after 4 h of chilling treatment, followed by a slight decrease after 8 h in both WT and *OE* plants; however, the activity was higher in *OE* plants than in WT plants (Fig. 5b). APX activity was lower in *OE* plants than in WT plants after 4 h of chilling treatment (Fig. 5c).

Under heat stress, the variation trends of SOD, CAT and APX activities were similar in the WT and transgenic plants. SOD showed significantly higher activities in the transgenic lines than in the WT after 4 and 8 h of heat stress (Fig. 5a). The respective activities of CAT in *OE2* and *OE5* were 2.23- and 2.79-fold higher than that measured in the control group after 4 h of heat treatment and 2.28- and 2.39-fold higher after 8 h of heat treatment, compared with increases of only 1.88- and 1.41-fold in the WT (Fig. 5b). APX activities were lower in transgenic plants than in WT plants exposed to heat stress (Fig. 5c).

The antioxidant enzyme activities were also measured in T1 generations of transgenic plants. After 8 h of chilling or heat treatment, higher SOD and CAT activities were measured in transgenic plants than in WT (Additional file 4: Figure S4B and D). The higher activities of SOD and CAT in *OE* plants under both chilling and heat conditions indicated that overexpression of sha-miR319d resulted in higher antioxidant activity in tomato.

#### Overexpression of sha-miR319d alters the expression of chilling-, heat- and ROS signaling genes

**Fig. 6:**
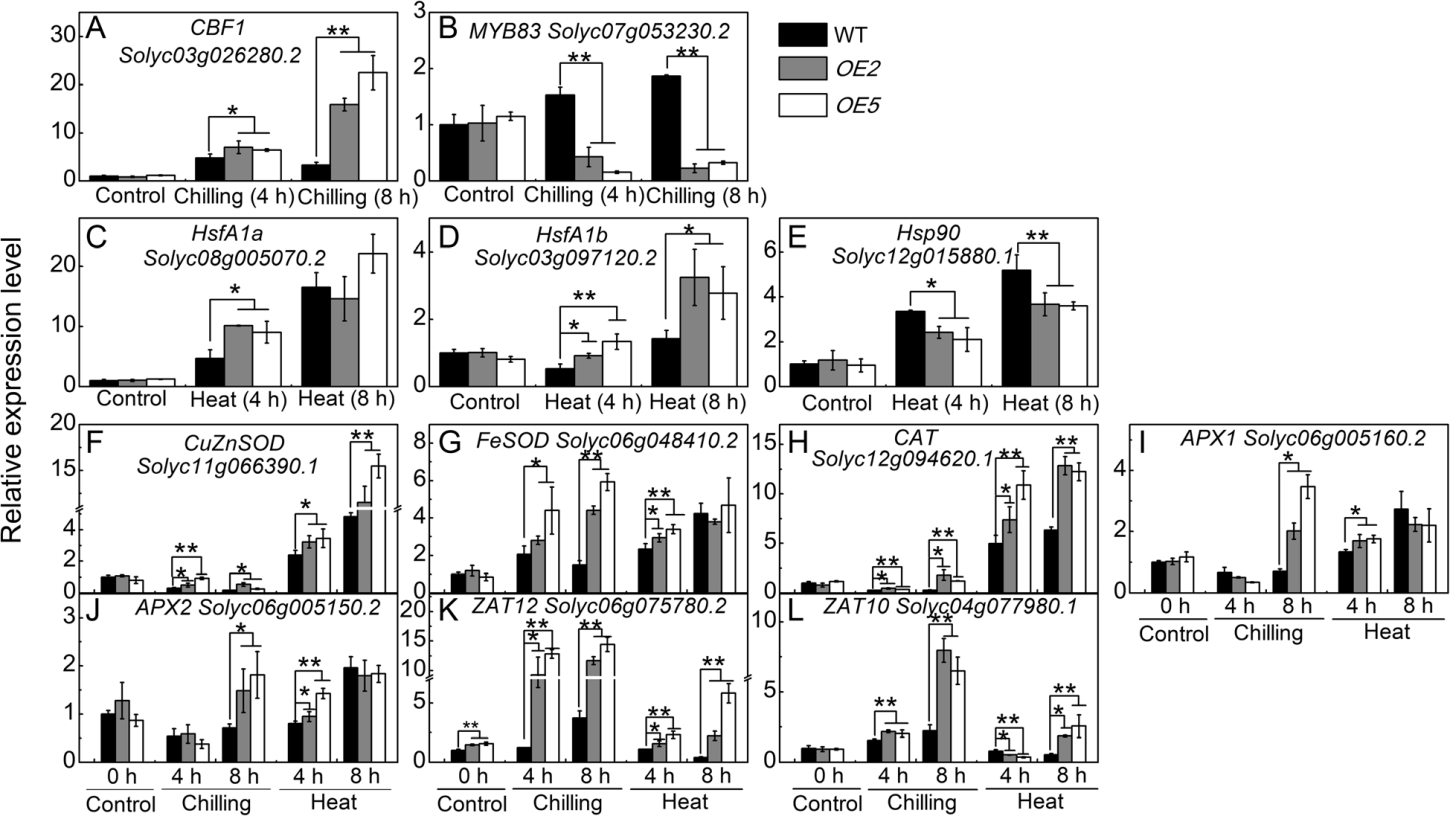
Relative expression levels of key genes involved in chilling, heat and ROS signaling. qPCR analysis of *CBF1* (**a**), *MYB83* (**b**), *HsfA1a* (**c**), *HsfA1b* (**d**), *Hsp90* (**e**), *CuZnSOD* (**f**), *FeSOD* (**g**), *CAT* (**h**), *APX1* (**i**), *APX2* (**j**), *ZAT12* (**k**) and *ZAT10* (**l**) in sha-miR319d-overexpressing lines and WT. T2 generations of transgenic plants as well as WT were treated with chilling or heat for 0 (control), 4 and 8 h. The reference gene was *Actin*. Each value is the mean of three biological repeats ± the standard deviation (SD). Asterisks indicate significant differences between WT and transgenic plants. *, *P* < 0.05; **, *P* < 0.01, Student’s t test.

The expression level of *CBF1* (*Solyc03g026280.2*) was significantly up-regulated in both WT and *OE* plants after chilling treatment; however, the increase in *OE* plants was much greater than that observed in WT (Fig. 6a). The expression of *MYB83* (*Solyc07g053230.2*), an *Arabidopsis MYB15* homolog and a negative regulator of the low temperature signaling pathway [14, 45], was markedly reduced in *OE* plants but was induced in WT plants (Fig. 6b). The expression levels of HsfA1 coding genes *HsfA1a* (*Solyc08g005170.2*) and *HsfA1b* (*Solyc03g097120.2*) were also increased more strongly in *OE* plants than in WT plants (Fig. 6c and d). By contrast, the transcription of Hsp90, which plays a role as a negative regulator of HsfA1 [20], was lower in transgenic plants than in WT plants after 4 and 8 h of heat stress (Fig. 6e). Altered expression of *CBF1, MYB83, HsfA1a, HsfA1b* and *Hsp90* were also observed in T1 generations (Additional file 5). These results provided evidence that overexpression of sha-miR319d affected the chilling and heat stress signal transduction pathways.

We further analyzed expression of *CuZnSOD* (*Solyc11g066390.1*), *FeSOD* (*Solyc06g048410.2*), *CAT* (*Solyc12g094620.1*), *APX1* (*Solyc06g005160.2*), and *APX2* (*Solyc06g005150.2*). Under normal conditions, there was no notable difference between WT and *OE* plants (Fig. 6f-j). Chilling stress repressed the expression of *CuZnSOD,* but the *OE* plants maintained higher expression levels than WT (Fig. 6f). Expression of *FeSOD* was strongly induced by chilling stress in *OE2* (2.55- and 5.36-fold compared with the control) and *OE5* (5.22- and 5.26-fold compared with the control) after 4 and 8 h of treatment, respectively, which was higher than that of WT (2.16- and 1.47-fold compared with the control) (Fig. 6g). A significantly higher level of *CAT* expression under chilling stress was also observed in *OE* plants (Fig. 6h). The expression levels of *APX1* and *APX2* were higher than those in WT only after 8 h of chilling treatment (Fig. 6i and j). Under heat stress, expression of *CuZnSOD* in *OE* plants was significantly higher after 4 and 8 h of treatment compared with the control group (Fig. 6f). Expression of *FeSOD* in *OE* plants was significantly higher after 4 h of treatment (Fig. 6g), and that of *CAT* in *OE* plants was also significantly higher after 4 and 8 h of treatment compared with the control group (Fig. 6h). The altered expression levels of antioxidant enzymes coding genes were almost in line with the enzyme activities, suggesting that the effect of sha-miR319d on antioxidant enzyme activities is through altered expression of their coding genes.

The C2H2 zinc finger transcription factor genes *ZAT12* (*Solyc06g075780.2*) and *ZAT10* (*Solyc04g 077980.1*), which are reported to be involved in multiple abiotic stresses and might serve as central roles in reactive oxygen signaling [14, 46, 47], were also analyzed by qPCR. As shown in Fig. 6k, expression of *ZAT12* in *OE* plants exhibited stable high levels under control, chilling, and heat conditions. No significant difference in *ZAT10* was observed in *OE* plants and WT under normal conditions, while it was higher in *OE* plants after 4 and 8 h of chilling and 8 h of heat treatment and was lower in *OE* plants after 4 h of heat treatment (Fig. 6l). Similar expression patterns of *CuZnSOD*, *FeSOD*, *CAT*, *ZAT12* and *ZAT10* in T1 generations are presented in Additional file 5. To confirm the results further, the "qPCR analysis of the relative expression levels of key genes involved in chilling, heat and ROS signaling" experiment was performed for the fourth biological repeat in T2 generations (Additional file 9) and similar expression patterns were obtained.

#### Sha-miR319d functions in plant tolerance to chilling stress possibly by inhibiting *GAMYB-like1* expression in tomato

**Fig. 7:**
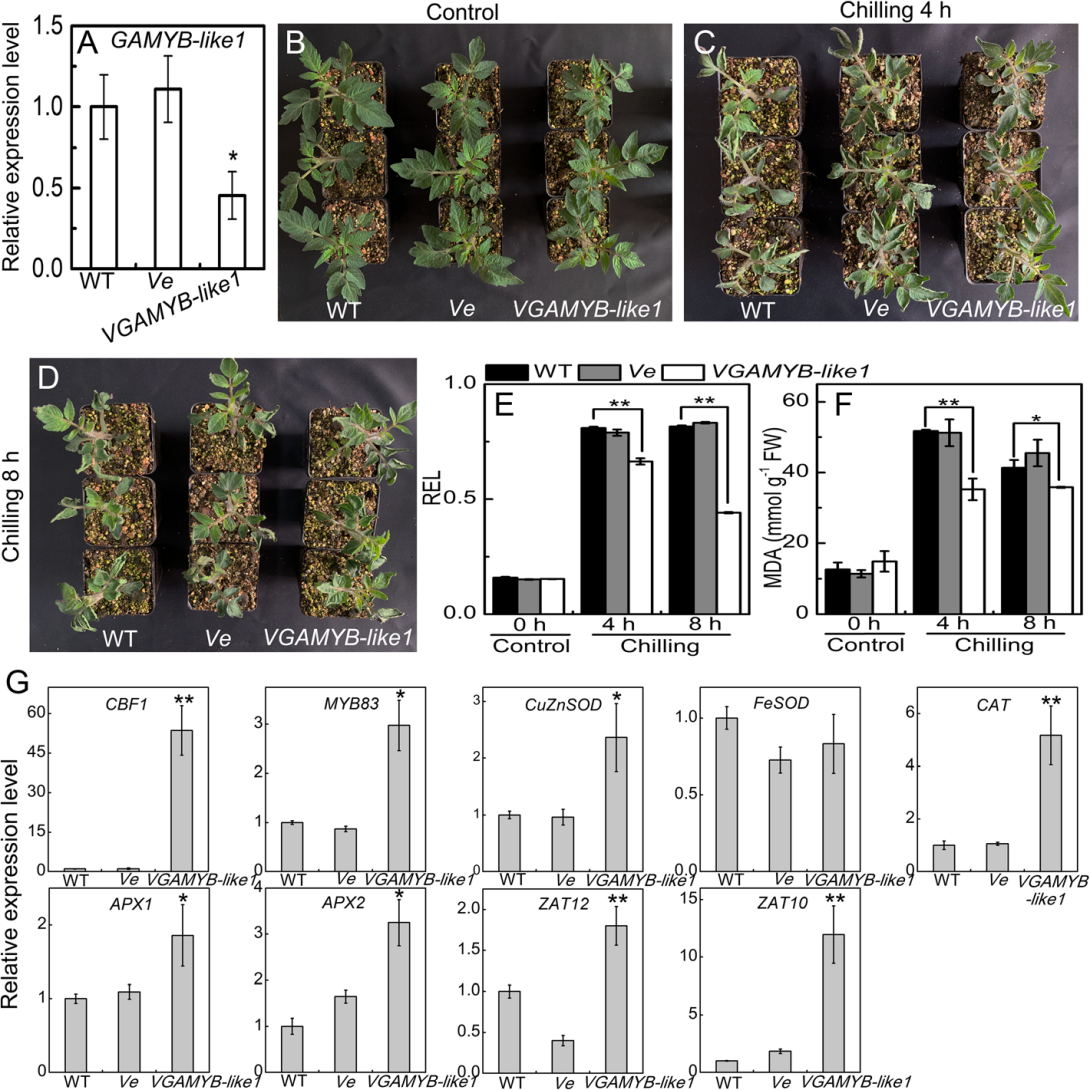
The effect of *GAMYB-like1*-silencing on tomato seedlings under chilling stress. **a** Relative expression levels of *GAMYB-like1* in WT, *Ve* and *VGAMYB-like1* plants. **b** Phenotypes of WT, *Ve* and *VGAMYB-like1* lines under control (25 °C) conditions. **c** Phenotypes of WT, *Ve* and *VGAMYB-like1* lines under chilling (4 °C) conditions for 4 h; **d** Phenotypes of WT, *Ve* and *VGAMYB-like1* lines under chilling (4 °C) conditions for 8 h. **e** and **f** Effects of chilling stress on REL (**e**) and MDA (**f**) in WT, *Ve* and *VGAMYB-like1* lines. **g** Relative expression levels of key genes involved in chilling and ROS signaling. The data are means of three biological repeats ± the standard deviation (SD). Asterisks indicate significant differences between gene-silencing lines and WT. *, *P* < 0.05; **, *P* < 0.01, Student’s test

The expression level of *GAMYB-like1* was decreased in *S. habrochaites* ‘LA1777’ under chilling stress (Additional file 6), corresponding to an increase in sha-miR319d expression. This result suggested that sha-miR319d regulated tomato chilling tolerance by inbibiting *GAMYB-like1* epression. Temperature stress assays showed that silencing of *GAMYB-like1* increased chilling tolerance in tomato (Fig. 7). Under chilling (4 °C) treatment for 4 h or 8 h, relative electrolyte leakage and MDA concentrations were lower in *GAMYB-like1*-silenced plants (*VGAMYB-like1*) than in WT and pTRV2-empty control (*Ve*) plants. The altered relative expression levels of *CBF1*, *CuZnSOD*, *CAT*, *APX1*, *APX2*, *ZAT12* and *ZAT10* in *VGAMYB-like1* were similar to those in sha-miR319d-overexpressing plants under chilling stress treatment. These results indicated that sha-miR319d participated tomato chilling stress regulation by modulating expression of *GAMYB-like1*. The effects of *GAMYB-like1* silencing on tomato under heat stress were also analyzed, though no significant change in phenotype was observed between WT and *GAMYB-like1*-silenced plants (Additional file 7).

## Discussion

### The role of miR319 in regulating tomato tolerance to temperature stress

There are three genes encoding CBFs in the tomato genome, and *CBF1* is induced in response to chilling stress [48]. Overexpression of tomato *CBF1* in *Arabidopsis* is sufficient to induce constitutive expression of CBF target genes and enhance chilling tolerance in transgenic plants [14, 15], suggesting that tomato has a functional CBF response pathway. Overexpression of *Arabidopsis CBF1* enhances the resistance of tomato leaves to chilling stress by maintaining the photochemical functions of PSII and PSI [49]. Oil palm *EgCBF3* confers stress tolerance in transgenic tomato plants [50]. Array-based transcript profiling results showed that a *CBF* was upregulated and that 63 MYB TFs were differentially expressed in Micro-Tom tomato after 14 days of storage at 5 °C [51]. In addition, by binding to promoters of CBF genes, MYB proteins are involved in the regulation of cold responses in plants [52], suggesting that CBF also plays a role in ‘Micro-Tom’ chilling stress.

Apart from direct pathway of the CBF regulon, several signaling molecules also affect *CBF1* expression in tomato, such as nitric oxide, hydrogen peroxide, methyl jasmonate, salicylic acid, gibberellin, and ethylene [53–58], and the increased chilling tolerance obtained by modulating pathways in tomato was accompanied by elevated *CBF1* expression levels. These results suggest the complex mechanism of the CBF regulon and signaling molecule interactions in plants under chilling stress. A previous study highlighted that the enhancement of chilling tolerance in transgenic tomato overexpressing *CBF1* may be due to induction of *CAT1* gene expression and activity of the enzyme [59]. In our present study, *CBF1* expression levels were increased and ROS accumulation and activities of antioxidant enzymes were altered in transgenic tomato overexpressing sha-miR319d, suggesting that the elevated *CBF1* expression was related with ROS signaling. Furthermore, overexpression of sha-miR319d resulted in higher chilling tolerance in transgenic tomato than in the WT, suggesting that the positive role of sha-miR319d in regulating tomato chilling tolerance may be accounted for by the up-regulated expression of *CBF1* and increased expression of oxidative stress-responsive genes to protect the plants from chilling stress.

The plant heat stress response includes complex and diverse system cooperation. HsfA1 is one of the major transcription factors that regulate the expression levels of genes encoding important Hsfs and further regulates expression of heat stress-inducible genes [22, 60]. The activity of HsfA1 is reported to be affected by many components, including Hsp90 (repressed HsfA1 activity) and several miRNAs [22]. In our present study, under heat stress, the expression levels of *HsfA1a*, *HsfA1b* and *Hsp90* were increased in both transgenic plants and the WT, whereas *HsfA1a* and *HsfA1b* increased more in transgenic plants than in the WT, and *Hsp90* exhibited the inverse pattern, suggesting that overexpression of sha-miR319d enhanced heat tolerance by up-regulating *HsfA1a* and *HsfA1b* under heat stress.

### The regulatory mechanisms of sha-miR319d in tomato temperature tolerance

ROS are generated and accumulate under various stresses, including chilling and heat stress [61]. In our present study, transgenic plants showed less ROS accumulation than WT plants, and the activities of SOD and CAT were significantly higher in transgenic plants, suggesting that the increased tolerance of transgenic plants might, at least partially, result from high activities of SOD and CAT leading to less ROS. Furthermore, the up-regulated expression levels of *FeSOD*, *CuSOD* and *CAT* in transgenic plants provided evidence that sha-miR319d had a positive effect on the antioxidant system under temperature stress at the transcriptional level.

In addition to oxidative damage, the role of ROS as signal transduction molecules has been analyzed for several years [22, 62, 63]. *ZAT12* and *ZAT10* are reported to play key roles in modulating expression of ROS-response genes. *ZAT12* can be induced by chilling, heat, salinity, drought and wounding in *Arabidopsis* [46]. In our present study, expression of *ZAT12* was elevated in transgenic plants (Fig. 6k), and that of *ZAT10* also differed from that in WT, suggesting that ROS signal transduction was changed by overexpression of sha-miR319d.

The involvement of ROS in temperature stress regulation has been demonstrated in several studies. For instance, *ZAT12* downregulated *CBF* transcript expression [64], *ZAT10*-overexpressing lines and *ZAT10*-RNAi lines have enhanced tolerance to abiotic stress [47], *HsfA4a* is induced by oxidative stress [46], and ROS are thought to serve as heat signaling molecules [65]. The loss-of-function of *apx1* and *cat2* mutants show heat-sensitive phenotypes [66]. These studies suggest a link between ROS and the temperature signaling pathway. Hence, the higher levels of *CBF1*, *Hsf1Aa* and *Hsf1Ab* in sha-miR319d transgenic plants under chilling or heat conditions might result in altered ROS signals. However, the components of the ROS signaling pathways, the mechanism of ROS chilling or heat signaling and the intensive role of sha-miR319d in signal transduction are largely unknown; therefore, extensive studies are needed.

**Fig. 8:**
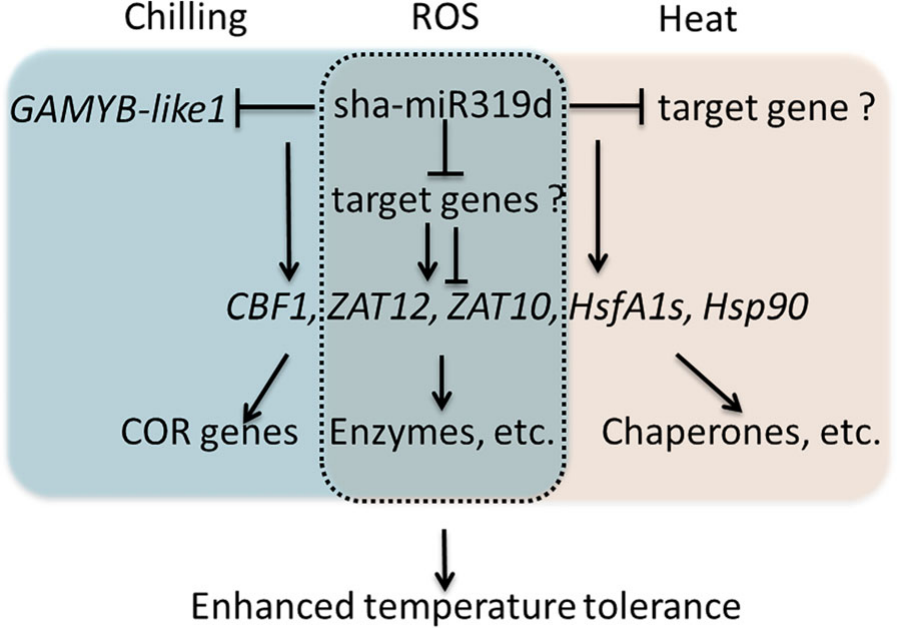
A possible regulatory model of sha-miR319d in tomato temperature stress. Arrows indicate positive regulation, and T bars indicate negative regulation

A possible regulatory model for sha-miR319d in tomato temperature stress is proposed based on our results. As shown in Fig. 8, low temperature and high temperature induce expression of sha-miR319d. Under chilling stress, sha-miR319d promotes expression of *CBF1*, *ZAT12* and *ZAT10*, possibly by inhibiting expression of *GAMYB-like1*. Under heat stress, sha-miR319d inhibits expression of *HsfA1a* and *ZAT12*, and inhibits that of *Hsp90.* Subsequently, the low temperature-response gene, high temperature-response gene and antioxidant-related gene are regulated and expressed to modulate the temperature tolerance and stress system of tomato.

### The potential role of sha-miR319d in tomato growth and stress tolerance regulation

In addition to its function in the plant stress response, the role of miR319 in growth regulation has been explored in numerous studies. In the present study, transgenic tomato lines generated by overexpressing the sha-miR319d precursor exhibited curly leaves and lower height compared with the WT, similar to previous reports [4, 5, 67, 68]. These results suggest that the function of miR319 in regulating leaf morphogenesis is conserved in various plants. A study on cucumber (*Cucumis sativus*) showed that two csa-miR393-mediated auxin receptors are also involved in leaf morphogenesis regulation, suggesting that miR319, miR393 and auxin signaling might have relationships with leaf development. Furthermore, although leaf morphogenesis was changed by overexpression of sha-miR319d, no difference was observed in the chlorophyll contents (Fig. 4a) and Fv/Fm value (Fig. 4b) between WT and *OE* lines under the control conditions (0 d of treatment), suggesting that overexpression of sha-miR319d did not decrease tomato photosynthesis under normal conditions.

In our present study, the putative targets of sha-miR319d showed significantly decreased expression in the sha-miR319d-overexpressing lines (Fig. S2), suggesting the regulatory effect of sha-miR319d on these target genes. Among these targets, *TCP1*, *TCP2*, *TCP3*, *TCP10*, *TCP24* and *TCP29* are *TCP* family members, which are involved in leaf, flower, and fruit development, as well as in hormonal stimulus responses [69–71]. *GAMYB-like1* is a R2R3 MYB family gene that has been suggested to be involved in germination [72] and chilling responses [40] and might be regulated by miR159 [40, 73]. We also explored it functional interactions using STRING software (https://string-db.org/). Two DEAD box helicase family members DEAH-6 and DEAH-21 were predicted to interact with GAMYB-like1 (Additional file 8), and several studies have demonstrated that DEAD box proteins participate widely in plant stress tolerance regulation [74–77]. Along with other putative unknown function targets, sha-miR319d targets are distributed across a wide range of plant growth and stress responses, suggesting the versatile roles of sha-miR319d. These data demonstrated that the potential genetic regulatory role of sha-miR319d can be utilized in future genetic improvement of tomato.

## Conclusions

In conclusion, sha-miR319d, a recently discovered member of the tomato miR319 family, positively regulates tomato temperature stress. Sha-miR319d can alter expression of several key genes (*CBF1, MYB83*) in chilling signal transduction, key genes (*HsfA1a*, *HsfA1b* and *Hsp90*) in heat signal transduction, and key genes (*ZAT12* and *ZAT10*) in ROS signal transduction and can promote the transduction of chilling, heat and ROS signals, thus improving the ability of transgenic tomatoes to withstand temperature stress. Silencing of *GAMYB-like1* increased tomato chilling tolerance, and the altered relative expression levels of *CBF1*, *CuZnSOD*, *CAT*, *APX1*, *APX2*, *ZAT12* and *ZAT10* in *VGAMYB-like1* plants were similar to those in sha-miR319d-overexpressing plants under chilling stress. These results indicate that sha-miR319d participates in tomato chilling stress regulation, possibly by regulating expression of *GAMYB-like1*. Under normal conditions, overexpression of sha-miR319d in tomato caused leaf crinkling and reduced plant height but had no effect on the chlorophyll content and Fv/Fm. The functions of the predicted target genes in multiple life processes suggested a stronger regulatory role of sha-miR319d in addition to temperature stress. Our research provides insight for further study of the role of sha-miR319d in tomato growth and stress regulation and lays a foundation for the genetic improvement of tomato.

## Methods

### Plant material and growth conditions

The wild tomato genotype, *S. habrochaites* ‘LA1777’, was used to isolate the precursor of sha-miR319d. The cultivated tomato ‘Micro-Tom’ wild-type (WT) was used as the transgenic acceptor in this study. The seeds of ‘LA1777’ were obtained from the Tomato Genetics Resource Center (TGRC, University of California, CA, US). The seeds of ‘MicroTom’ were kindly provided by Professor Jinfeng Chen (College of Horticulture, Nanjing Agricultural University, China). After soaking and pre-germination treatments, tomato seeds were sown in plastic plots and were raised in growth chambers (Dongnan Instrument, RDN-560E-4, China) set at 25/18 °C (14/10 h, day/night) with a light intensity of 280 ± 20 μmol m^− 2^ s^− 1^.

### Transformation of tomato plants

To generate sha-miR319d overexpression lines [78], a 396-bp DNA fragment harboring the sha-MIR319d hairpin structure was amplified using the primers sha-319-4-F and sha-319-4-R. The ‘LA1777’ genomic DNA was used as a PCR template. The fragments were cloned into the pMD19-T simple vector (Takara, China) prior to sequencing. Then, fragments were digested with BamHI (Thermo, US) and XhoI (Thermo, US) and were inserted into the corresponding sites of pENTR1A (Invitrogen, US) to generate an entry clone. Thereafter, pENTR1A containing sha-MIR319d was introduced into the pMDC32 vector with a 35S-caulifower mosaic virus promoter (CaMV35S, 35S) by LR recombination reactions (Invitrogen, US). The construct plasmid was transformed into *Agrobacterium tumefaciens* strain GV3101 and was further transformed into WT tomato using the *A. tumefaciens*-mediated leaf disk method according to Fillatti et al. [79]. Primary tomato transformants (T0) were identified by PCR using 35S promoter-specific forward primers together with sha-miR319d-specific reverse primers (Additional files 10).

### Temperature stress treatments

T2 transgenic generation lines (*OE2*, *OE5* and the WT) were used for chilling and heat stress assays (a portion of the T1 generation experimental results are shown in the supplementary materials). Approximately 30-day-old transgenic lines and WT seedlings were exposed to chilling stress (4 °C) or heat stress (40 °C) in the growth chambers. Each biological repeat contained five seedlings from every tomato genotype, and there were three biological repeats per treatment.

### Sample collections and index measuring

#### Measuring REL

REL was measured according to the method of Cao et al. [36]. After chilling or heat treatment for 0 and 1 d, 0.1 g fresh weight of leaf discs (5 mm in diameter) was collected in a 15-mL centrifuge tube containing 10 mL of distilled deionized water, vacuumed for 30 min and then stored at room temperature for 1 h. The initial electrolyte leakage (R1) was measured with a conductivity detector (DDSJ-308A, China) at 25 °C. Subsequently, the centrifuge tube was heated in boiling water for 20 min and then cooled to room temperature. The final electrolyte leakage (R2) was measured at 25 °C. REL was calculated as R1/R2.

#### Measuring the MDA content

After chilling or heat treatment for 0 and 1 d, approximately 0.3 g fresh weight of leaf sample was placed in 3 mL of 10% (m/v) trichloroacetic acid trichloroacetic acid (TCA). After centrifugation at 10,000 rpm for 15 min, 2 mL of the supernatant was mixed with 2 mL of 0.6% thiobarbituric acid [TBA, 0.6% (w/v) thiobarbituric acid in 10% TCA]. The mixture was heated in boiling water for 20 min and then cooled to room temperature. The absorbance of the supernatant after centrifugation at 10,000 rpm for 15 min was determined by spectrophotometry at 450, 532, and 600 nm. The formula C_MDA_ (μmol L^− 1^) = 6.45*(OD_532_-OD_600_)-0.56*OD_450_ was used to calculate the content of MDA.

#### Measuring the chlorophyll content

After chilling or heat treatment for 0 and 5 d, 0.1 g of fresh weight of leaf samples was ground with 5 mL of 80% acetone and centrifuged at 12,000 rpm for 10 min. Then, the supernatant was used to measure the chlorophyll content. The absorbance was determined at 649 and 665 nm. The total chlorophyll content was calculated according to the formula in Cao et al. [36].

#### Measuring Fv/Fm

Fv/Fm was measured using a plant efficiency analyzer (MINI-PAM-II, WALZ, Germany). The measurement was performed at 0, 1, 2, 3 and 4 d after chilling or heat treatment. The seedlings were adapted to the dark for 20 min before being measured. The third fully expanded leaf of each plant was used in this measurement. In total, 24 seedlings per line were measured at one time point per treatment.

#### Histochemical staining of O_2_^−^ and H_2_O_2_

O_2_^−^ was detected visually by treating leaves with NBT as described by Rao and Davis [80]. After chilling or heat treatment for 0 and 1 d, leaf samples were put into a centrifuge tube containing 0.5 mg mL^− 1^ NBT (Sigma, US) supplied with 10 mM sodium phosphate buffer (pH 7.8) and was vacuumed for 30 min and then incubated overnight at 25 °C. To remove the green color, the leaves were de-stained by 75% (v/v) ethyl alcohol in an 85 °C water bath. The samples were photographed after cooling. The concentration of H_2_O_2_ was measured by DAB staining according to the method of Giacomelli et al. [81]. Leaf samples were placed into a 1 mg mL^− 1^ DAB solution (pH 3.8) and then incubated, de-stained and photographed following the method described above.

#### Measuring O_2_^−^ generation and the H_2_O_2_ content

The O_2_^−^ generation rate was measured using the hydroxylamine oxidization method described by Wang and Luo [82]. Briefly, after chilling or heat treatment for 1 d, 0.1 g of leaf sample was ground with 1 mL of cold 50 mM phosphate buffer (pH 7.8) in a cold mortar. After centrifugation at 12,000 rpm for 10 min at 4 °C, 0.5 mL of supernatant was added to 0.5 mL 50 mM phosphate buffer (pH 7.8) and 1 mL 1 mM hydroxylamine chloride and incubated for 1 h at 25 °C. Then, 1 mL of 17 mM p-aminobenzene sulfonic acid and 1 mL of 7 mM α-naphthylamine were added and incubated for 20 min at 25 °C after which the absorbance was measured at 530 nm.

The H_2_O_2_ concentration was measured according to the method of Uchida et al. [83]. After chilling or heat treatment for 0 and 1 d, 0.1 g of leaf sample was ground with 1 mL of cold 0.1% (m/v) trichloroacetic acid trichloroacetic acid (TCA) in an ice bath. After centrifugation at 12,000 rpm for 15 min at 4 °C, 0.5 mL supernatant was added to 0.5 mL 0.1 M phosphate buffer (pH 7.0) and 1 mL of 1 M potassium iodide (KI) and then incubated for 1 h at 25 °C in the dark. The absorbance was determined at 390 nm. The H_2_O_2_ content was calculated using a standard curve of known concentrations of H_2_O_2_.

#### Enzyme activity assays

Samples were collected at 0, 4 and 8 h after chilling or heat treatment. A 0.1 g leaf sample was ground with 3 mL of cold 50 mM phosphate buffer (pH 7.0) in a cold mortar and was then transferred to a centrifuge tube. The homogenates were centrifuged at 4 °C for 15 min at 12,000 rpm. The supernatant was used for the assay for the activities of antioxidant enzymes. SOD was assayed by monitoring its ability to inhibit the photochemical reduction of NBT according to the method of Beyer and Fridovich [84]. One unit of SOD activity was defined as the amount of enzyme required for 50% inhibition of NBT reduction, which was monitored at 560 nm. CAT activity was determined by potassium (KMnO_4_) permanganate titration in accordance with Li [85]. CAT activity was defined as the amount of H_2_O_2_ decomposed per minute. APX activity was determined by monitoring the decrease in absorbance at 290 nm due to ascorbate oxidation by H_2_O_2_, according to the method of Jimenez et al. [86]. One unit of APX activity was defined as an absorbance decrease of 0.01 per minute at 290 nm.

#### qPCR analysis

Total RNA was isolated from plant material using TRIzol reagent (Bioteke, China). One microgram of total RNA was used for reverse transcription using the PrimeScript RT reagent kit (Takara, China) according to the manufacturer’s recommendations. For miRNA synthesis, stem-loop reverse transcription RT primers for sha-miR319d (Additional file 10) were designed according to the criteria described by Tang et al. [87].

Real-time qPCR was performed using SYBR Premix Ex Taq (Takara, China) in a Quantstudio3 real-time PCR machine (Applied Biosystems, US) according to the manufacturer’s instructions. Tomato U6 small nuclear RNA (*U6snRNA*) and *Actin* [29] were used as the reference genes of miRNAs and mRNAs, respectively. The qPCR primer sequences of *TCP1*, *TCP10*, *TCP24*, *GAMYB-like1*, *DUF761*, *Kelch-type3*, *PPR-containing*, *HsfA1a*, *HsfA1b*, *ZAT12* and *ZAT10* were referenced from the qPrimerDB database (https://biodb.swu.edu.cn/qprimerdb/) [88]. The qPCR primer sequence of *Hsp90* was referenced from Liu et al. [89]. The primers used in this study are shown in Additional file 10. The qPCR reactions were repeated three times per sample, and the expression levels were calculated by the 2^−ΔΔCT^ method [90].

#### Silencing of *GAMYB-like1* genes by VIGS

A tobacco rattle virus (TRV)-based vector, including pTRV1 and pTRV2 VIGS vectors, was used in this experiment. For constructing pTRV2-GAMYB-like1, a 426-bp *GAMYB-like1* cDNA fragment corresponding to bases 439–938 bp of tomato the *GAMYB-like1* gene was amplified from tomato ‘MicroTom’ cDNA by PCR using the primers VGA1F and VGA1R. The PCR product was cloned into BamHI-XhoI-digested pTRV2. The plasmid was transformed into *Agrobacterium tumefaciens* strain GV3101.

Gene silencing by VIGS was performed according to the procedures of described by Liu et al. [91, 92], with some modifications. *Agrobacterium* containing pTRV1, pTRV2 or pTRV2-GAMYB-like1 was grown overnight at 28 °C in yeast extract peptone (YEP) medium supplemented with 50 mg L^− 1^ kanamycin and 50 mg L^− 1^ rifampicin. The next day, the culture was inoculated into 50 mL YEP medium supplemented with 50 mg L^− 1^ kanamycin, 50 mg L^− 1^ rifampicin, 10 mM MES and 20 μM acetosyringone. *Agrobacterium* cells were harvested and resuspended in infiltration medium (10 mM MgCl_2_, 10 mM MES, 200 μM acetosyringone), adjusted to an OD_600_ of 1.5. A mixture of *Agrobacterium* cultures containing pTRV1 and pTRV2-GAMYB-like1 or pTRV2 (empty vector control) at a ratio of 1:1 (v/v) were placed at 25 °C for 4 h before infiltration into the cotyledons of ‘MicroTom’ using a needleless 1-mL syringe. The plants were left covered overnight.

At approximately 1 month after infiltration, the seedlings were used for chilling and heat stress assays. Temperature treatments and index measuring were determined according to the methods described above.

#### Phylogenetic analysis

Phylogenetic analysis was carried out using the maximum likelihood method based on the Tamura and Nei [93] model. The analysis involved sha-MIR319d and 8 other homologous nucleotide sequences. All sequences were obtained from miRBase22.0 [42]. Evolutionary analyses were conducted in MEGA5 [94]. Target gene prediction was carried out using the psRNA Target tool [43].

### Statistical analysis

Data points are the means ± the standard deviation [38] of three biological replicate samples. Each biological replicate sample is composed of at least five individual seedling leaves. The statistical significance of differences among WT and transgenic plants was analyzed by Student’s t-tests. Asterisks * and ** indicate significant differences at *P* < 0.05 and *P* < 0.01, respectively. The data for seedling height are the means ± SD of 15 seedlings. The data for Fv/Fm are the means ± SD obtained from 24 seedlings, and the statistical significance of differences was analyzed by analysis of variance (ANOVA) using SPSS 18.0. Figures were drawn using Origin 9.0.

## Additional files

Additional file 1: **Figure S1.** Sequence analysis of sha-miR319d. Phylogenetic analysis of sha-MIR319d and its homologs and target gene prediction. A Sequence comparison between sha-miR319d and potato (*S. tuberosum*) stu-miR319d (miRBase 22 accession number: MIMAT0031276). **B** Molecular phylogenetic analysis by the Maximum Likelihood method based on the Tamura-Nei model. Evolutionary analyses were conducted in MEGA5. (TIF 2505 kb)

Additional file 2: **Figure S2** Sha-miR319d target gene prediction and expression level analysis. A Sha-miR319d target genes were predicted using the psRNATarget tool. **B** Expression levels of putative target genes in WT and T1 generations of sha-miR319d-overexpressing transgenic plants. The reference genes was *Actin.* Each value is the mean of three biological repeats ± the standard deviation (SD). Asterisks indicate significant differences between WT and transgenic plants. *, *P* < 0.05; **, *P* < 0.01, Student’s t test. (TIF 2498 kb)

Additional file 3: **Figure S3** Identification of sha-miR319d-overexpressing transgenic tomatoes and the effect of sha-miR319d overexpression on plants. **A** Expression levels of sha-miR319d in WT and T1 generations of transgenic plants. **B** Phenotypes of 25-day-old WT and transgenic lines (T1 generations) under normal (25 °C) conditions. **C** Phenotypes of WT and transgenic lines (T1 generations) under control (25 °C) conditions. **D** Phenotypes of WT and transgenic lines (T1 generations) under chilling (4 °C) conditions for 2 d. **E** Phenotypes of WT and transgenic lines (T1 generations) under heat (40 °C) conditions for 2 d. The reference genes for sha-miR319d was *U6snRNA*. Each value is the mean of three biological repeats ± the standard deviation (SD). Asterisks indicate significant differences between WT and transgenic plants. *, *P* < 0.05; **, P < 0.01, Student’s t test. (TIF 20646 kb)

Additional file 4: **Figure S4** The effect of overexpression of sha-miR319d on ROS accumulation and related enzyme activities**. A** NBT staining for O_2_^−^ in the leaves of WT and transgenic plants under control conditions and after 1 d of chilling (4 °C) or heat (40 °C) treatment. **B** DAB staining for H_2_O_2_ in the leaves of WT and transgenic plants under control conditions and after 1 d of chilling (4 °C) or heat (40 °C) treatment. **C** SOD activities of WT and transgenic plants under control, chilling (4 °C) and heat stress (40 °C). **D** CAT activities of WT and transgenic plants under control, chilling (4 °C) and heat stress (40 °C). The T1 generations of transgenic plants as well as WT were used. Each value is the mean of three biological repeats ± the standard deviation (SD). Asterisks indicate significant differences between transgenic lines and WT. *, P < 0.05; **, P < 0.01, Student’s t test. Bars in (A) and (B) indicate 1 cm. (TIF 9935 kb)

Additional file 5: **Figure S5** Relative expression levels of key genes in chilling, heat and ROS signaling. qPCR analysis of *CBF1* (A), *MYB83* (B), *HsfA1a* (C), *HsfA1b* (D), *Hsp90* (E), *CuZnSOD* (F), *FeSOD* (G), *CAT* (H), *ZAT12* (I) and *ZAT10* (J) in sha-miR319d-overexpressing lines and WT. T1 generations of transgenic plants as well as WT were treated with chilling or heat for 0 (control) and 8 h. The reference gene was *Actin*. Each value is the mean of three biological repeats ± the standard deviation (SD). Asterisks indicate significant differences between WT and transgenic plants. *, *P* < 0.05; **, *P* < 0.01, Student’s t test. (TIF 402 kb)

Additional file 6: **Figure S6.** Relative expression levels of *GAMYB-like1* under chilling stress in *S. habrochaites* ‘LA1777’. The reference gene was *Actin*. Each value is the mean of three biological repeats ± the standard deviation (SD). Different letters above indicate statistical significance of differences (P < 0.05) analyzed by variance (ANOVA) using SPSS 18.0. (TIF 118 kb)

Additional file 7: **Figure S7.** Phenotype of WT and GAMYB-like1-silenced plants under heat stress. B Phenotypes of WT, *Ve* and *VGAMYB-like1* lines under control (25 °C) conditions. **C** Phenotypes of WT, *Ve* and *VGAMYB-like1* lines under heat (40 °C) conditions for 4 h; D. Phenotypes of WT, *Ve* and *VGAMYB-like1* lines under heat (40 °C) conditions for 8 h. (TIF 17944 kb)

Additional file 8: **Figure S8**. Functional interaction of GAMYB-like1. (TIF 2551 kb)

Additional file 9: **Figure S9.** Relative expression levels of key genes involved in chilling, heat and ROS signaling (fourth biological repeat). qPCR analysis of *CBF1* (A), *MYB83* (B), *HsfA1a* (C), *HsfA1b* (D), *Hsp90* (E), *CuZnSOD* (F), *FeSOD* (G), *CAT* (H), *ZAT12* (I) and *ZAT10* (J) in sha-miR319d-overexpressing lines and WT. The reference gene was Actin. Each value is the mean of three technical repeats ± the standard deviation (SD). (TIF 473 kb)

Additional file 10: **Table S1**. Primers used in this study (DOCX 20 kb)

## Abbreviations

APX: ascorbate peroxidase
CAT: catalase
DAB: 3,3-diaminobenzidine
NBT: Nitro blue tetrazolium
qPCR: Quantitative real-time PCR
SOD: Superoxide dismutase
VIGS: Virus-induced gene silencing
WT: Wild-type
