# Supplementary figures and images for: Overexpression of Solanum habrochaites microRNA319d (sha-miR319d) confers chilling and heat stress tolerance in tomato (S. lycopersicum)
Source: BMC Plant Biol. 2019 May 23;19:214. doi: 10.1186/s12870-019-1823-x (PMC6533698; doi:10.1186/s12870-019-1823-x)

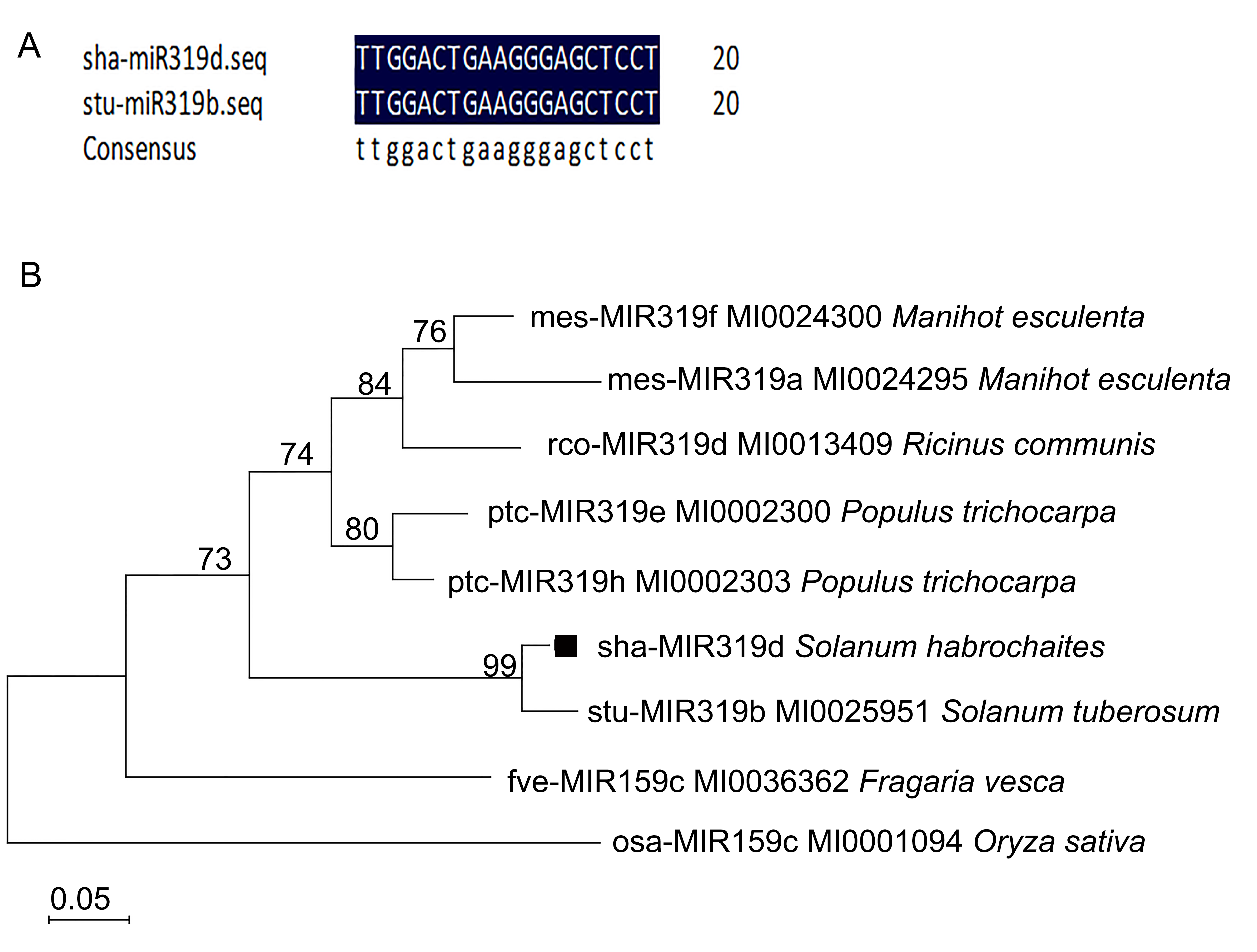

Supplement: Supplementary file 1 — Figure S1. Sequence analysis of sha-miR319d. Phylogenetic analysis of sha-MIR319d and its homologs and target gene prediction. A Sequence comparison between sha-miR319d and potato (S. tuberosum) stu-miR319d (miRBase 22 accession number: MIMAT0031276). B Molecular phylogenetic analysis by the Maximum Likelihood method based on the Tamura-Nei model. Evolutionary analyses were conducted in MEGA5. (TIF 2505 kb) [file 12870_2019_1823_MOESM1_ESM.tif]

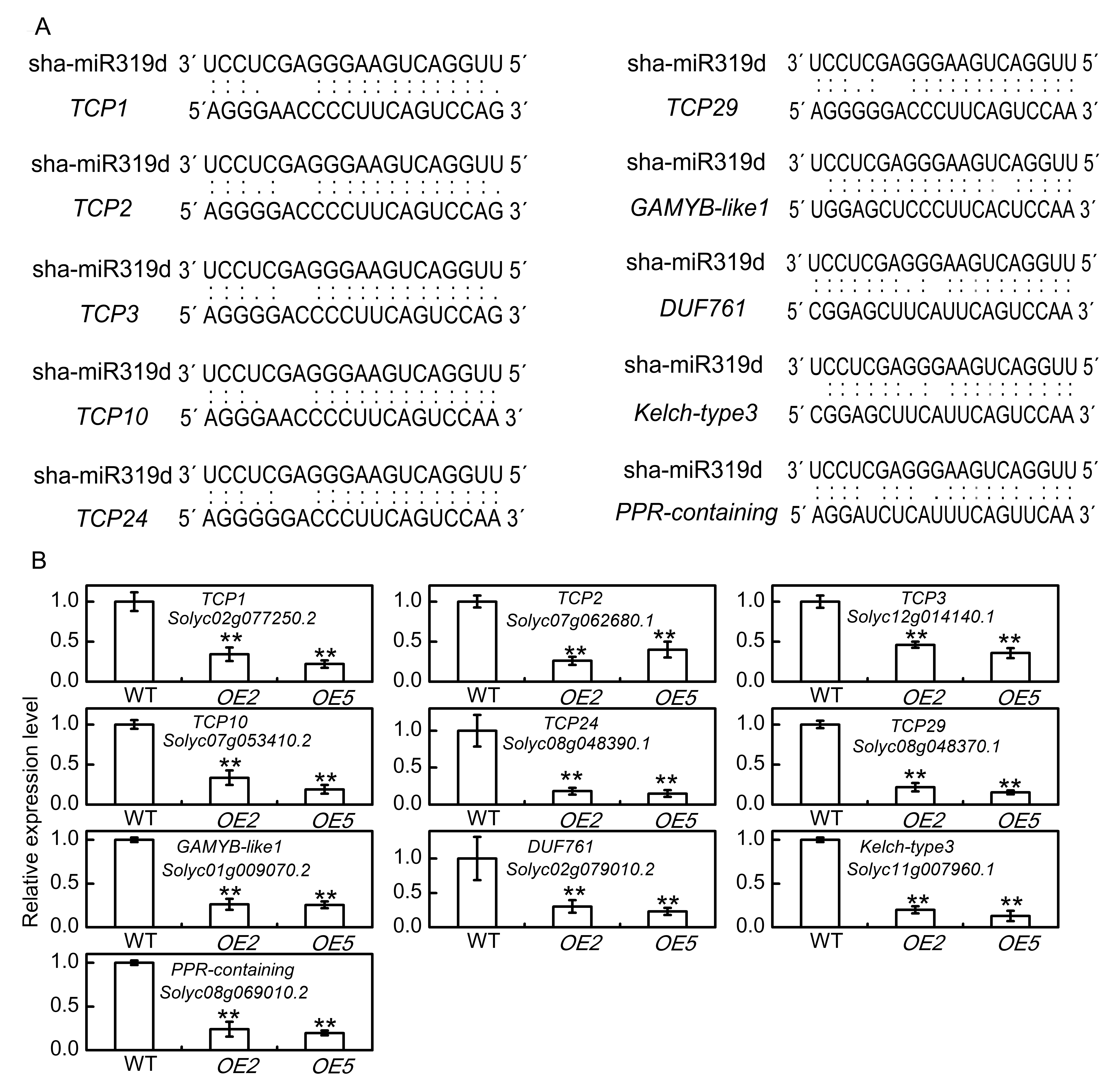

Supplement: Supplementary file 2 — Figure S2 Sha-miR319d target gene prediction and expression level analysis. A Sha-miR319d target genes were predicted using the psRNATarget tool. B Expression levels of putative target genes in WT and T1 generations of sha-miR319d-overexpressing transgenic plants. The reference genes was Actin. Each value is the mean of three biological repeats ± the standard deviation (SD). Asterisks indicate significant differences between WT and transgenic plants. *, P < 0.05; **, P < 0.01, Student’s t test. (TIF 2498 kb) [file 12870_2019_1823_MOESM2_ESM.tif]

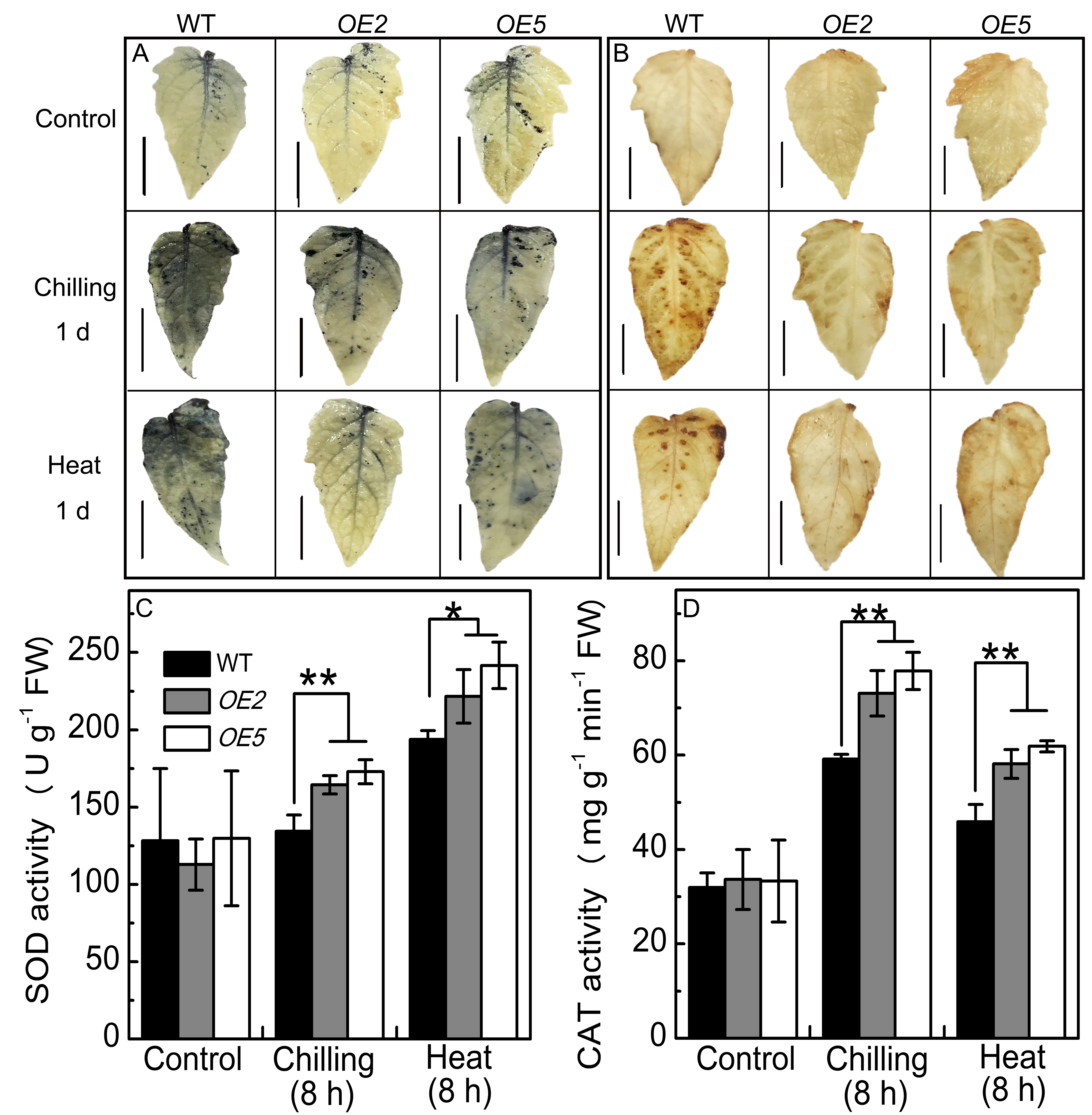

Supplement: Supplementary file 4 — Figure S4 The effect of overexpression of sha-miR319d on ROS accumulation and related enzyme activities. A NBT staining for O2− in the leaves of WT and transgenic plants under control conditions and after 1 d of chilling (4 °C) or heat (40 °C) treatment. B DAB staining for H2O2 in the leaves of WT and transgenic plants under control conditions and after 1 d of chilling (4 °C) or heat (40 °C) treatment. C SOD activities of WT and transgenic plants under control, chilling (4 °C) and heat stress (40 °C). D CAT activities of WT and transgenic plants under control, chilling (4 °C) and heat stress (40 °C). The T1 generations of transgenic plants as well as WT were used. Each value is the mean of three biological repeats ± the standard deviation (SD). Asterisks indicate significant differences between transgenic lines and WT. *, P < 0.05; **, P < 0.01, Student’s t test. Bars in (A) and (B) indicate 1 cm. (TIF 9935 kb) [file 12870_2019_1823_MOESM4_ESM.tif]

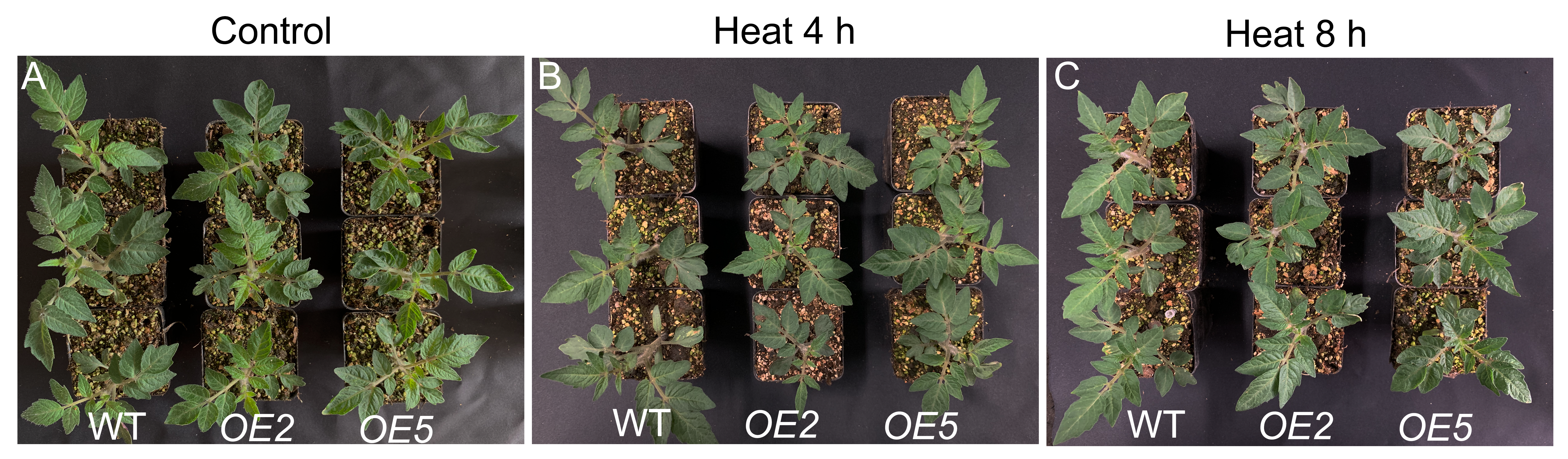

Supplement: Supplementary file 7 — Figure S7. Phenotype of WT and GAMYB-like1-silenced plants under heat stress. B Phenotypes of WT, Ve and VGAMYB-like1 lines under control (25 °C) conditions. C Phenotypes of WT, Ve and VGAMYB-like1 lines under heat (40 °C) conditions for 4 h; D. Phenotypes of WT, Ve and VGAMYB-like1 lines under heat (40 °C) conditions for 8 h. (TIF 17944 kb) [file 12870_2019_1823_MOESM7_ESM.tif]

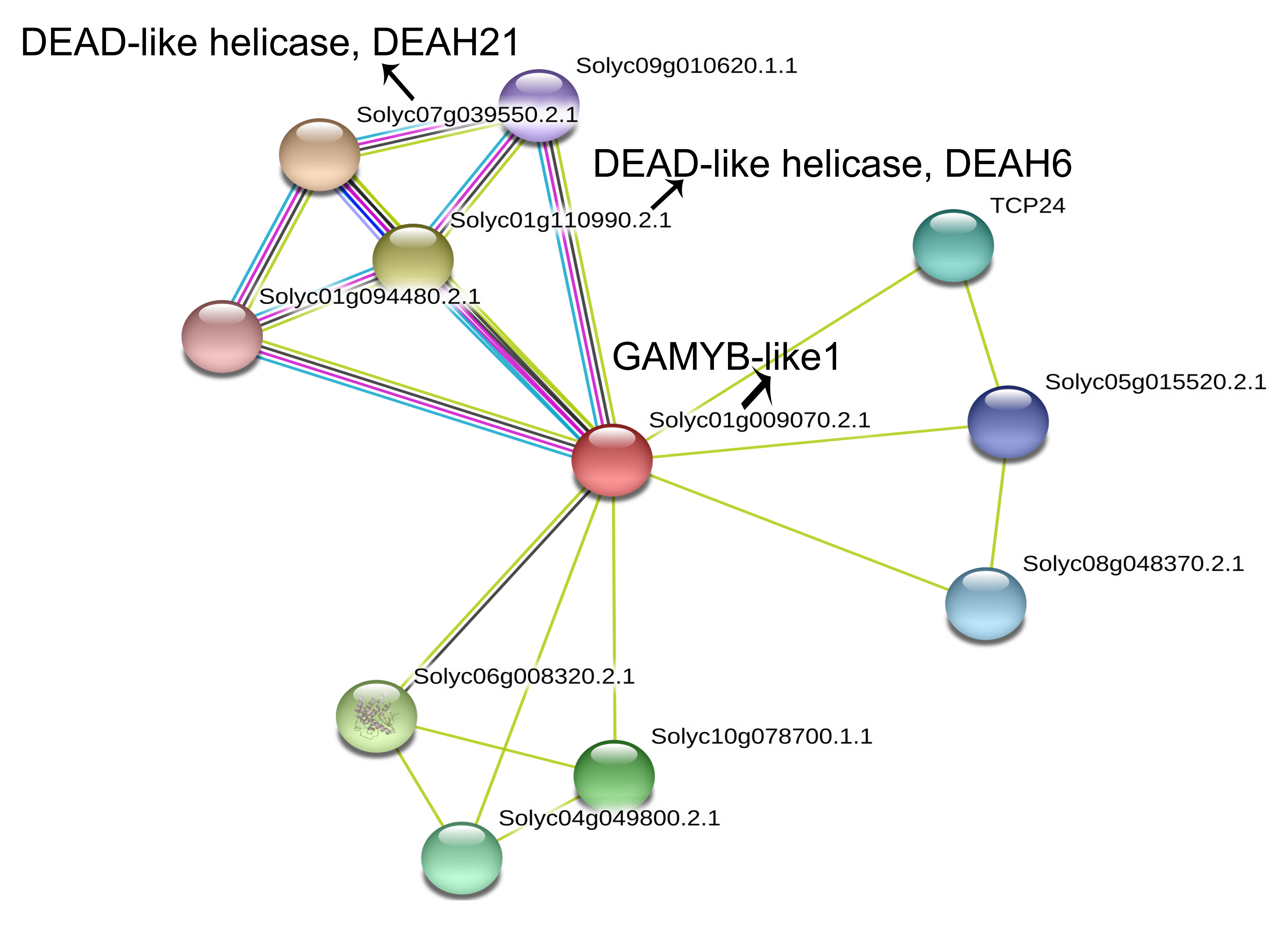

Supplement: Supplementary file 8 — Figure S8. Functional interaction of GAMYB-like1. (TIF 2551 kb) [file 12870_2019_1823_MOESM8_ESM.tif]
