# Supplementary material for: Overexpression of Solanum habrochaites microRNA319d (sha-miR319d) confers chilling and heat stress tolerance in tomato (S. lycopersicum)
Source: BMC Plant Biol. 2019 May 23;19:214. doi: 10.1186/s12870-019-1823-x (PMC6533698; doi:10.1186/s12870-019-1823-x)
Supplement: Supplementary file 10 — Table S1. Primers used in this study (DOCX 20 kb) [file 12870_2019_1823_MOESM10_ESM.docx]

**Additional file 10:** Tab. S1 Primers used in this experiment

| Primer | Sequence (5´to 3´) | Application |
| --- | --- | --- |
| miR319d stem-loop RT primer | GTCGTATCCAGTGCGTGTCGTGGAGTCGGCAATTGCACTGGATACGACAGGAGCT | RT-PCR of miR319d |
| U6snRNA stem-loop RT primer | GTGCAGGGTCCGAGGTTTTGGACCATTTCTCGAT | RT-PCR of *U6snRNA* |
| miR319d F | GTAAGGAGACTATGCCAAGC | qPCR of miR319d |
| 3’ universal primer | CAGTGCGTGTCGTGGAGT |  |
| U6snRNA-F | GGAACGATACAGAGAAGATTAGCA | Reference gene of sha-miR319d qPCR |
| U6snRNA-R | GTGCAGGGTCCGAGGT |  |
| sha-319-4-F | GGATCCATATAAATAGCTGATGGAAGTGAAG | pMDC32- sha-MIR319d vector construction |
| sha-319-4-R | CTCGAGGCTAGAACAAGCTAAGGTTAAAATT |  |
| 35SU | GAAGGTGGCTCCTACAAATGCCA | Identification of transgenic plants |
| TCP1F | CTGCAAAAAGCCAGAAAAATGG | qPCR of *TCP1* |
| TCP1R | TACTGTGTCTGTCTTTCCTTCC |  |
| TCP2F | CAGCAGCTATTCGGTCAAAATCAGT | qPCR of *TCP2* |
| TCP2R | GCCAGGAATTGTTGATGGATACATT |  |
| TCP3F | AAATCAGTTTTTGTCTCAGAGGGGA | qPCR of *TCP3* |
| TCP3R | CGATGCCAGAAAGTGATGTTGAATA |  |
| TCP10F | TCCAAGAATGGCTAGTTGGAAT | qPCR of *TCP10* |
| TCP10R | CTAGCTGGCACTTGAAAATCAG |  |
| TCP24F | AGAAGTCAAAAAGAGTAGCGGA | qPCR of *TCP24* |
| TCP24R | CTATGCCTATCTTTCCCTCCAG |  |
| TCP29F | GACCAGTGTCTCCACCAATGTTTAG | qPCR of *TCP29* |
| TCP29R | GGAACCAAGATATGATCCGTACCTC |  |
| GAMYB-like1F | CAATTTCCTTAGCGATGGTAGC | qPCR of *GAMYB-like1* |
| GAMYB-like1R | AACCAAAAGGATCTTTCGAAGC |  |
| DUF761F | CAGCAGCTATTCGGTCAAAATCAGT | qPCR of *DUF761* |
| DUF761R | CAAAGTAGAGATACGGAGGCTT |  |
| Kelch-type3F | GTCTAAGGAGCTTCAGTCAGTT | qPCR of *Kelch-type3* |
| Kelch-type3F | GGACTGCATAGACTCTAGTGTC |  |
| PPR-containingF | TGTGTTGGAATACTGCATTGTG | qPCR of *PPR-containing* |
| PPR-containingR | CTGTTCAGACGTAAAAACTCCG |  |
| CBF1F | ATCATCTTCTTCTTCATCGTCATC | qPCR of *CBF1* |
| CBF1R | TCCTTGTCTTCTTATTTGGTTCTC |  |
| MYB83F | TGCAATTCACTTACCAGGAAGA | qPCR of *MYB83* |
| MYB83R | CACTTGAGTTATAATGAGCGCG |  |
| HsfA1aF | AGCGGTGATCGATATTTGTTTG | qPCR of *HsfA1a* |
| HsfA1aR | GTAGGATTCTTCGCATTGCAAT |  |
| HsfA1bF | TTGTCTCTGATGAATTTTCGGC | qPCR of *HsfA1b* |
| HsfA1bR | GAGATCCTCTTCCACGACTAAG |  |
| Hsp90F | GCACTTCTCTGTTGAAGGTCAG | qPCR of *Hsp90* |
| Hsp90R | ATGAACACACGGCGAACATA |  |
| CuZnSODF | TCTTCACCACAACCAGCACT | qPCR of *CuZnSOD* |
| CuZnSODR | CAGTAAGGGGTTTAGGGGTAGT |  |
| FeSODF | GGGAAGCATCACAGGGCGTATG | qPCR of *FeSOD* |
| FeSODR | GGCTCTCCTCCTCCGTTGG |  |
| CATF | TCCTTGTCGTCCTGCTGAG | qPCR of *CAT* |
| CATR | TTGATGTATCTGTCTTGCCTGTC |  |
| APX1F | TGCTGGTACCTACGATGTGTG | qPCR of *APX1* |
| APX1R | CTGGTGGCTCTGGCTTGTC |  |
| APX2F | GGCTGGTGTTGTTGCTGTTG | qPCR of *APX2* |
| APX2R | TCAGGCAAGCGACCTTCAAC |  |
| ZAT12F | GCCATCGAACGAGTCATAAATC | qPCR of *ZAT12* |
| ZAT12R | CCTGACCCATAGAAAACTCCAT |  |
| ZAT10F | GCAAACGTTTCAATTTGAGAGC | qPCR of *ZAT10* |
| ZAT10R | CGTGACCTCTTAGATCTCTTCC |  |
| ActinF | GAAATAGCATAAGATGGCAGACG | Reference gene of qPCR |
| ActinR | ATACCCACCATCACACCAGTAT |  |
| VGA1F | GGATCCATGGCAAGTCAGAACAAACAAAATG | pTRV2-GAMYB-like1 vector construction |
| VGA1R | CTCGAGTGCTCGCTGATCATCATATGTTAAA |  |

Note: Added restriction enzyme sites are underlined.
